# Silica-Triggered Autoimmunity in Lupus-Prone Mice Blocked by Docosahexaenoic Acid Consumption

**DOI:** 10.1371/journal.pone.0160622

**Published:** 2016-08-11

**Authors:** Melissa A. Bates, Christina Brandenberger, Ingeborg I. Langohr, Kazuyoshi Kumagai, Adam L. Lock, Jack R. Harkema, Andrij Holian, James J. Pestka

**Affiliations:** 1 Department of Food Science and Human Nutrition, Michigan State University, East Lansing, MI, 48824, United States of America; 2 Institute for Integrative Toxicology, Michigan State University, East Lansing, MI, 48824, United States of America; 3 Institute of Functional and Applied Anatomy, Hannover Medical School, Hannover, 30625, Germany; 4 Department of Pathobiological Studies, School of Veterinary Medicine, Louisiana State University, Baton Rouge, LA, 70803, United States of America; 5 Department of Pathobiology and Diagnostic Investigation, Michigan State University, East Lansing, MI, 48824, United States of America; 6 Department of Animal Science, Michigan State University, East Lansing, MI, 48824, United States of America; 7 Center for Environmental Health Sciences, University of Montana, Missoula, MT, 59812, United States of America; Instituto Nacional de Ciencias Medicas y Nutricion Salvador Zubiran, MEXICO

## Abstract

Occupational exposure to respirable crystalline silica (cSiO_2_, quartz) is etiologically linked to systemic lupus erythematosus (lupus) and other human autoimmune diseases (ADs). In the female NZBWF1 mouse, a widely used animal model that is genetically prone to lupus, short-term repeated intranasal exposure to cSiO_2_ triggers premature initiation of autoimmune responses in the lungs and kidneys. In contrast to cSiO_2_’s triggering action, consumption of the ω-3 polyunsaturated fatty acid docosahexaenoic acid (DHA) prevents spontaneous onset of autoimmunity in this mouse strain. The aim of this study was to test the hypothesis that consumption of DHA will prevent cSiO_2_-triggered autoimmunity in the female NZBWF1 mouse. Mice (6 wk old) were fed isocaloric AIN-93G diets containing 0.0, 0.4, 1.2 or 2.4% DHA. Two wk after initiating feeding, mice were intranasally instilled with 1 mg cSiO_2_ once per wk for 4 wk and maintained on experimental diets for an additional 12 wk. Mice were then sacrificed and the lung, blood and kidney assessed for markers of inflammation and autoimmunity. DHA was incorporated into lung, red blood cells and kidney from diet in a concentration-dependent fashion. Dietary DHA dose-dependently suppressed cSiO_2_-triggered perivascular leukocyte infiltration and ectopic lymphoid tissue neogenesis in the lung. DHA consumption concurrently inhibited cSiO_2_–driven elevation of proinflammatory cytokines, B-cell proliferation factors, IgG and anti-dsDNA Ig in both bronchoalveolar lavage fluid and plasma. DHA’s prophylactic effects were further mirrored in reduced proteinuria and glomerulonephritis in cSiO_2_-treated mice. Taken together, these results reveal that DHA consumption suppresses cSiO_2_ triggering of autoimmunity in female NZBWF1 mice as manifested in the lung, blood and kidney. Our findings provide novel insight into how dietary modulation of the lipidome might be used to prevent or delay triggering of AD by cSiO_2_. Such knowledge opens the possibility of developing practical, low-cost preventative strategies to reduce the risk of initiating AD and subsequent flaring in cSiO_2_-exposed individuals. Additional research in this model is required to establish the mechanisms by which DHA suppresses cSiO_2_-induced autoimmunity and to ascertain unique lipidome signatures predictive of susceptibility to cSiO_2_-triggered AD.

## Background

Autoimmune diseases (ADs) affect 24 million individuals in the U.S., inflicting tremendous individual suffering and societal costs [1]. The genome is universally recognized to be a primary predisposing factor for AD; however, the exposome, an individual’s lifetime environmental exposures, is a contributor of considerable and underappreciated importance. One component of the exposome likely to be critical to AD onset and progression is exposure to respirable particles such as crystalline silica (cSiO_2,_ quartz) [2]. An estimated 2.3 million Americans employed in construction, manufacturing, mining, hydraulic fracturing, farming and custodial service are exposed to high concentrations of cSiO_2_ [3]. These individuals are at increased risk of developing ADs that include systemic lupus erythematosus (lupus), rheumatoid arthritis, scleroderma and antineutrophil cytoplasmic antibody-associated vasculitis [4–7].

Lupus, a prototypical AD, is characterized by loss of tolerance to self-antigens, activation of autoreactive B and T cells and consequential production of pathogenic autoantibodies [8]. These antibodies complex with self-antigens (e.g. nuclear components, including dsDNA) and deposit in tissues facilitating mononuclear cell infiltration. In the kidney, these events drive onset and progression of glomerulonephritis—a burdensome and potentially fatal complication of lupus. Mouse strains that spontaneously develop lupus have been widely employed to understand how toxic stressors impact AD pathogenesis [9]. One example is the NZM2410 mouse, which develops lupus nephritis early in life causing them to succumb by 25 wk of age [10]. Intranasal cSiO_2_ instillation of NZM2410 accelerates the onset of lupus-related responses comprising of elevated autoreactive antibodies, proteinuria, and glomerulonephritis [11–14]. Accordingly, both experimental animal and human epidemiological studies suggest that cSiO_2_ could be a significant environmental trigger for lupus and potentially other ADs.

The female New Zealand Black White (F1) mouse (NZBWF1), another widely used murine model of human lupus [9], spontaneously shows autoantibody production early in life. Glomerulonephritis onset is observed at approximately 34 wk of age, resulting in very high mortality by 12 months of age. Our laboratory recently demonstrated that weekly intranasal exposure to cSiO_2_ for 4 wk beginning at 9 wk of age of NZBWF1 mice dramatically shortened glomerulonephritis onset time to 22 wk of age [15]. Concurrently, cSiO_2_ elicited intense inflammatory responses in the lungs as evidenced by robust perivascular and peribronchial lymphocytic cell infiltration. These immune cells consisted of large numbers of B (CD45R^+^) cells, T (CD3^+^) cells and IgG-secreting plasma cells indicative of ectopic lymphoid tissue (ELT) neogenesis. Consistent with pulmonary inflammation and ELT development, bronchiolar lavage fluid (BALF) from cSiO_2_-exposed mice displayed elevated concentrations of IgG and the cytokines MCP-1, TNF-α and IL-6. cSiO_2_ exposure also evoked increases in plasma autoantibodies and proinflammatory cytokines. The discovery that cSiO_2_-induced inflammation and ELT neogenesis within the pulmonary compartment of NZBWF1 mice occurs in parallel with systemic inflammatory and autoimmune responses strongly suggests the lung functions as a platform for AD triggering by this respirable particle.

Another component of an individual’s exposome that potentially influences susceptibility to autoimmunity is diet [16]. Fat is a macronutrient that prominently influences immune function, with dietary polyunsaturated fatty acids (PUFA) being of particular relevance [17]. PUFA that contain more than one double bond in their backbone are classified as ω-3 or ω-6 according to the position of the first double bond relative to the terminal carbon in the aliphatic chain. Generally, ω-6 PUFA are regarded as proinflammatory in nature, whereas ω-3 PUFA such as eicosapentaenoic acid (EPA) and docosahexaenoic acid (DHA) are recognized to be anti-inflammatory. Linoleic acid (C18:2 ω-6; LA), the major PUFA found in food oils derived from plants (e.g. corn and soybean), is the primary PUFA found in Western diets. Following consumption by humans, LA is enzymatically elongated and desaturated to arachidonic acid (C20:4 ω-6; ARA). Both LA and ARA become major components of cell membranes throughout the body. Although the ω-3 PUFA α-linolenic acid (C18:3 ω-3; ALA) is also found in plant oils, humans and other mammals have limited capacity to elongate it to eicosapentaenoic acid (C20:5 ω-3; EPA) and docosahexaenoic acid (22:6 ω-3; DHA) and afterwards incorporate these metabolites into cell membranes [18]. Thus, humans require exogenous sources for ω-3 PUFA other than plants or terrestrial animals. Certain marine microalgae efficiently catalyze the formation of EPA and DHA and cold-water fish consuming such algae readily bioconcentrate these ω-3 PUFA into their tissue [19]. Consequently, fish, fish oil and, more recently, microalgal oil are important sources of EPA and DHA for humans [20]. Animal and clinical studies over the past 30 years indicate that ingestion of ω-3 PUFA both prevents and resolves inflammation and, hence, might help those individuals who have a genetic predisposition to AD [21]. With the exception of multivitamins, ω-3 PUFA are the most widely used nutritional supplement [22, 23]. An estimated 30 million American adults regularly consume ω-3 PUFA suggesting both wide usage and consumer acceptance; however, the impact of dose on potential health effects remains unclear.

In a prior study, we compared latency and severity of autoimmunity in NZBWF1 mice consuming 1) an ω-3 PUFA- diet containing DHA-enriched fish oil, 2) an ω-6 PUFA-rich Western-type diet containing corn oil or 3) an ω-9 monounsaturated fatty acid (MUFA)-rich Mediterranean-type diet containing high oleic safflower oil [24]. Spontaneous elevations of plasma autoantibodies, proteinuria and glomerulonephritis occurred in mice at 34 wk of age fed ω-6 PUFA or ω-9 MUFA diets; however, these endpoints were dramatically diminished or even blocked in mice fed the DHA-rich diet. Inhibition of autoimmunity in ω-3 PUFA-fed NZBWF1 mice was associated with generalized downregulation of genes in both the kidney and spleen that contribute to inflammatory responses, antigen presentation, T cell activation, B cell activation/differentiation and leukocyte recruitment. Notably, many of these genes are currently under consideration as biomarkers and/or biotherapeutic targets for lupus.

Nothing is known about the interactive effects cSiO_2_ exposure and ω-3 consumption on the development of AD. To address this knowledge gap, we tested the hypothesis that consumption of DHA-rich microalgal oil will prevent cSiO_2_-triggered autoimmunity in the female NZBWF1 mouse. The results show that DHA supplementation dose-dependently suppressed most indicators of cSiO_2_-accelerated autoimmune pathogenesis in lung, blood and kidney in this lupus-prone mouse model.

## Materials and Methods

### Animals and diets

The Institutional Animal Care and Use Committee at Michigan State University reviewed and approved all animal procedures in accordance with National Institutes of Health guidelines (AUF# 01/12-002-00). Female 6 wk old NZBWF1 and control NZW/LacJ mice were obtained from Jackson Laboratories (Bar Harbor, ME). Upon arrival, mice were randomized into experimental groups. They were then housed four per cage with free access to food and water. Animals were maintained at constant temperature and humidity (21°C–24°C and 40–55%, respectively) under a 12 h light/dark cycle.

Formulations of experimental diets are summarized in Table 1. Briefly, modified American Institute of Nutrition (AIN)-93G diet containing 70 g/kg fat was prepared as described previously [24, 25]. All diets contained 10 g/kg food-grade corn oil to provide basal essential fatty acids. Control diet (CON) was formulated with 60 g/kg food-grade high-oleic safflower oil (Hain Pure Foods, Boulder, CO). High-oleic safflower oil was replaced with 10, 30, or 60 g/kg microalgal oil containing 40% DHA (DHASCO^TM^, provided by Dr. Kevin Hadley, Martek Biosciences Corporation Columbia, MD) yielding experimental diets that corresponded to 0.4, 1.2, or 2.4% (w/w) DHA, respectively. Based on energy percentages, these concentrations are estimated to mimic human consumption of 2, 6, and 12 g/d of DHA, respectively. Fatty acid compositions based on total lipids in each diet were analyzed as described below and are summarized in Table 2.

**Table 1:** Compositions of experimental diets.

	Experimental Group			
	CON	0.4% DHA	1.2% DHA	2.4% DHA
Ingredient	(g/kg)			
Casein	200	200	200	200
Dyetrose	132	132	132	132
Cornstarch	397.5	397.5	397.5	397.5
Sucrose	100	100	100	100
Cellulose	50	50	50	50
t-Butylhydroquinone (TBHQ)	0.01	0.01	0.01	0.01
AIN 93G Salt Mix	35	35	35	35
AIN 93G Vitamin Mix (with vitamin E)	10	10	10	10
L-cysteine	3	3	3	3
Choline Bitartrate	2.5	2.5	2.5	2.5
Corn Oil^a^^,^^b^	10	10	10	10
High-Oleic Safflower Oil^a^^,^^c^	60	50	30	0
DHA-Enriched Algal Oil^a^^,^^d^	-	10	30	60

^a^ As reported by the manufacturer

^b^ Corn oil contained 612 g/kg linoleic acid and 26 g/kg oleic acid

^c^ High oleic safflower oil contained 750 g/kg oleic acid and 140 g/kg linoleic acid

^d^ Algal oil contained 395 g/kg DHA and 215 g/kg oleic acid

**Table 2:** Fatty acid content of experimental diets based GLC analysis.

Experimental Group				
	CON	0.4% DHA	1.2% DHA	2.4% DHA
Fatty Acid	(% of fatty acid of total lipid in the diet)			
16:0	8.31 ± 0.05	8.94 ± 0.14	10.1 ± 0.04	12.1 ± 0.06
16:1	0.12 ± 0.00	0.21 ± 0.01	0.37 ± 0.005	0.65 ± 0.01
18:0	2.61 ± 0.02	2.58 ± 0.03	2.47 ± 0.01	2.25 ± 0.02
18:1 *trans*	0.05 ± 0.00	0.05 ± 0.00	0.06 ± 0.00	0.06 ± 0.00
18:1 *cis*	46.4 ± 0.35	42.6 ± 0.90	35.6 ± 0.43	22.3 ± 0.10
18:2 (ω-6)	36.6 ± 0.29	36.1 ± 0.46	34.5 ± 0.29	32.2 ± 0.47
18:3 (ω-3)	3.15 ± 0.03	3.25 ± 0.06	3.26 ± 0.04	3.22 ± 0.04
20:4 (ω-6)	0.02 ± 0.00	0.02 ± 0.00	0.02 ± 0.00	0.02 ± 0.00
20:5 (ω-3)	0.0 ± 0.00	0.0 ± 0.00	0.01 ± 0.00	0.01 ± 0.00
22:5 (ω-3)	0.0 ± 0.00	0.03 ± 0.00	0.09 ± 0.00	0.19 ± 0.00
22:6 (ω-3)	0.0 ± 0.00	2.47 ± 0.30	7.74 ± 0.17	17.6 ± 0.52
∑ SFA	12.1 ± 0.07	13.7 ± 0.26	16.8 ± 0.09	22.4 ± 0.12
∑ MUFA	47.8 ± 0.36	44.0 ± 0.90	37.1 ± 0.43	24.0 ± 0.10
∑ PUFA (ω-3)	3.15 ± 0.03	5.75 ± 0.34	11.1± 0.18	21.0 ± 0.50
∑ PUFA (ω-6)	36.6 ± 0.29	36.1 ± 0.46	34.6 ± 0.29	32.3 ± 0.47
(ω-6):(ω-3)	11.6:1	6.3:1	3.1:1	1.5:1

### Experimental design

**Fig 1:**
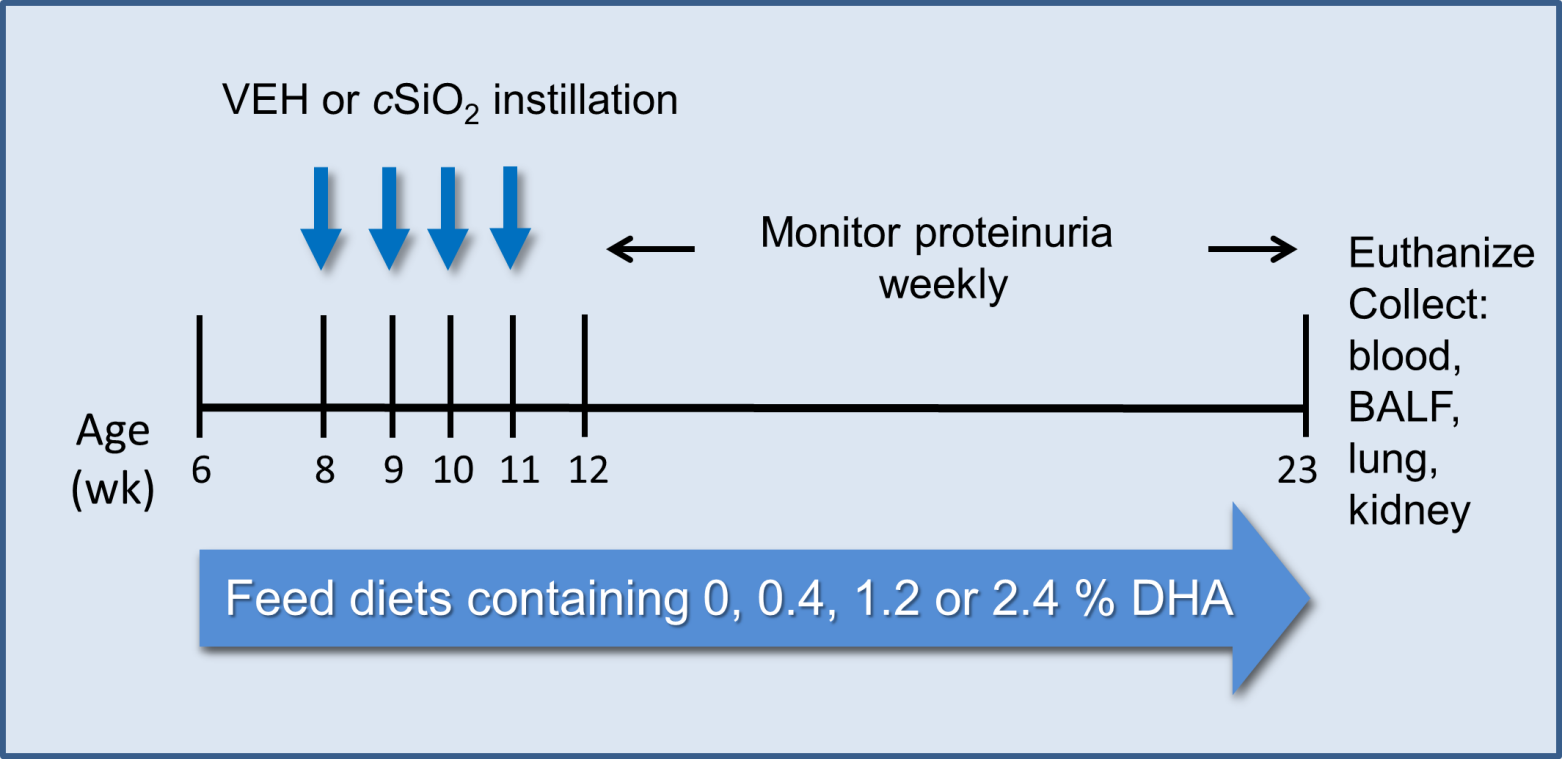
Experimental design. Beginning at age 6 wk, NZBWF1 were assigned CON diet or 0.4, 1.2, and 2.4% DHA diet. NZW/LacJ mice were assigned either CON diet or 2.4% DHA-containing diet. Then, starting at age 8 wk, mice were dosed intranasally with 25 μl PBS VEH or 25 μl PBS containing 1.0 mg cSiO_2_ weekly for 4 wk. Proteinuria was monitored over the course of the experiment and all animals euthanized at 23 wk of age (i.e. 12 wk PI).

Overall study design is depicted in Fig 1. Upon arrival, NZBWF1 mice (6 wk old) were fed CON diet or 0.4, 1.2, or 2.4% DHA-containing diets and maintained on assigned diet until experiment termination. To limit formation of lipid oxidation products, diets were prepared biweekly, stored at -20°C until use, and provided fresh to mice every other day. Beginning at 8 wk of age, mice were anesthetized with 4% isoflurane and intranasally instilled with 1.0 mg cSiO_2_ (Min-U-Sil-5, 1.5–2.0 μm average particle size, Pennsylvania Sand Glass Corporation, Pittsburgh, PA) in 25 μl PBS or PBS vehicle (VEH) once per wk for 4 wk [15]. The total dose of cSiO_2_ (4 mg) in mice approximates one half of a human lifetime exposure at the recommended occupational health guideline established by the Occupational Safety and Health Administration [3]. Urine was collected weekly and evaluated for protein using reagent dipsticks (Cortez Diagnostics, Inc., Calabasas, CA). Mice were euthanized and tissues collected 12 wk after the final cSiO_2_ exposure. At this time point, 3 out of 8 mice fed CON diet and exposed to cSiO_2_ displayed proteinuria (defined as > 300 mg/dL). Female NZW/LacJ mice were used as controls for selected endpoints; these are a parental line for the NZBWF1 hybrid that do not spontaneously develop lupus [10, 26],. Age-matched female NZW/LacJ mice were fed either CON or 2.4% DHA-enriched AIN-93G for 2 wk prior to vehicle or cSiO_2_ exposure and then maintained on experimental diets as described above.

### Necropsy and tissue collection

Mice were euthanized by intraperitoneal injection with 56 mg/kg BW sodium pentobarbital. Blood was collected from the abdominal aorta using heparinized syringes and centrifuged at 3500 x g for 10 min at 4°C. Resultant plasma was stored at -80°C. Red blood cell pellets were immediately frozen in liquid nitrogen and stored at -80°C for fatty acid analysis. Bronchoalveolar lavage fluid (BALF) was collected from whole lungs as described previously [27]. After lavage, the right lung lobes were removed, frozen in liquid nitrogen and stored at -80°C for fatty acid analysis. The left lung lobe was fixed with 10% neutral buffered formalin (Fisher Scientific, Pittsburgh, PA) at constant pressure (30 cm H_2_O) for minimum of 1 h and stored in fixative until further processing for histology. The right kidney was excised and the cranial portion fixed in 10% neutral buffered formalin for 24 h. The left kidney was removed and stored at -80°C for fatty acid analysis.

### Fatty acid analyses

Fatty acid concentrations of diets were determined using a modification of a direct transesterification method [28] as reported previously [29]. Total lipids were extracted from lung and kidney tissue according to the method of Bligh and Dyer [30] using a mixture of methanol:chloroform:water (2:2:1.8, by vol). Red blood cells were isolated and purified from whole blood according to Burton and coworkers [31] and lipids extracted as described for lung and kidney. Fatty acid methyl esters (FAME) were prepared from lipid extracts using KOH solution and boron trifluoride in methanol [32]. Gas liquid chromatography (GLC) analysis of FAME extracts were performed on a GC-2010 gas chromatograph (Shimadzu) equipped with a flame ionization detector using a CP-Sil 88 capillary column (100m × 0.25mm I.D. × 0.25 μm film thickness, Varian). Gas chromatographic conditions were as detailed previously [29]. Individual FAME were identified by comparison of retention times with known FAME standards (GLC reference standard 63-A and GLC reference standard 455 from Nu-Chek Prep Inc.). Fatty acid results are expressed as percentages (w/w) of fatty acids detected with a chain length between 10 and 24 carbon atoms. The lower limit of detection was <0.001 g/100 g fatty acids.

### Kidney histopathology

Formalin-fixed, paraffin-embedded kidneys were sectioned to 5 μm and stained with either hematoxylin and eosin (H&E) or Periodic Acid Schiff and hematoxylin (PASH). Slide sections were individually graded by a board-certified veterinary pathologist graded using a modified International Society of Nephrology-Renal Pathology Society Lupus Nephritis Classification [33] as follows: (0) no tubular proteinosis and normal glomeruli; (1) mild tubular proteinosis with multifocal segmental proliferative glomerulonephritis and occasional early glomerular sclerosis and crescent formation; (2) moderate tubular proteinosis with diffuse segmental proliferative glomerulonephritis, early glomerular sclerosis and crescent formation; and (3) marked tubular proteinosis with diffuse global proliferative and sclerosing glomerulonephritis.

### Lung histopathology

The formalin-fixed inflated left lung lobe was cut in randomly oriented 2 mm thick serial sections. All sections (4–6 per left lung lobe) were embedded in paraffin for histopathological and morphometric analysis. Tissue sections (5 μm) were routinely stained with H&E for cellular morphometry and histopathology. Sections were semi-quantitatively scored by a board-certified veterinary pathologist for the following lung lesions: (a) presence of lymphocytic cell infiltration within perivascular and peribronchial regions, (b) alveolitis defined as the presence of alveolar infiltration of vacuolated macrophages, neutrophils, and lymphocytes, granuloma formation in the alveolus, type II epithelial cell hyperplasia, and thickened alveolar wall, and (c) presence of alveolar proteinosis. Individual lungs were graded for these lesions using the following criteria (% of total pulmonary tissue examined): (0) no changes compared to control mice; (1) minimal (<10%); (2) slight (10–25%); (3) moderate (26–50%); (4) severe (51–75%); or (5) very severe (>75%) of total area affected.

### Lung immunohistochemistry

Immunohistochemistry was performed on formalin-fixed, paraffin embedded lung sections for identification of B and T lymphocytes in lung parenchyma. Briefly, B and T cells were identified using rat monoclonal anti-mouse CD45R (BD Biosciences, San Jose, CA) (1:300) and rabbit polyclonal anti-mouse CD3 (Abcam, Cambridge, MA) (1:100), respectively. Deparaffinized tissue sections (4 μm) were first subjected to heat-induced epitope retrieval with citrate buffer (pH 6.0) at 100°C for 30 min (CD45R) or 125°C for 15 min (CD3). Following pretreatments to block non-specific binding and endogenous biotin, primary antibody staining was performed. Bound CD45R and CD3 antibodies were detected by incubation with biotinylated secondary antibodies followed by Peroxidase Reagent and Nova Red substrate (Vector Laboratories, Inc., Burlingame, CA) prior to being counterstained with hematoxylin.

### Lung morphometry for B and T cells

To quantify B and T cell infiltration, slides stained with antibodies to CD45R and CD3, respectively, were scanned and digitized with a VS110 (Olympus, Hicksville, NY) virtual slide system. The lung tissues on scanned slides were selected as region of interest and 10% of the lung section was captured at 20X magnification by systematic random sampling with NewCast software (Visiopharm, Hoersholm, Denmark). The volume density of CD45R^+^ or CD3^+^ cells in the bronchial and perivascular regions of the lungs was estimated by projecting a point grid over the randomly sampled images with the STEPanizer 1.8 Stereology Tool (http://www.stepanizer.com/)[34]. The numbers of points hitting the CD45R^+^ or CD3^+^ cells as well as the whole lung tissues (i.e. lung parenchyma and interstitium) were counted. Finally, the volume density or percentage of CD45R^+^ and CD3^+^ per reference area was calculated.

### BALF cell quantitation and identification

Total cell numbers in BALF were determined by counting intact cells using a hemocytometer without Trypan Blue exclusion. Cytological slides from BALF were prepared by centrifugation at 400 x g for 10 min using a Shandon Cytospin 3 (Shandon Scientific, PA), allowed to air dry, and stained with Diff-Quick (Fisher Scientific). Remaining BALF was centrifuged at 2400 x g for 15 min and supernatant collected and stored at -80°C. Differential cell counts for macrophages/monocytes, lymphocytes, neutrophils, and eosinophils in BALF were determined using morphological criteria from 200 total cells on cytological slides.

### IgG and autoantibody measurement

Total IgG in BALF and plasma was determined by ELISA [35]. Briefly, 96-well plates were coated with 50 μL per well of 5 μg/ml goat anti-mouse IgG (γ-chain specific, Alpha Diagnostics, Inc., cat # 40120) in carbonate coating buffer. Afterwards, BALF and plasma samples as well as standards were loaded on the plate, followed by another incubation step with horseradish peroxidase-labeled goat anti-mouse IgG (γ-chain specific) (Alpha Diagnostics, Inc., cat # 40121). The standard curve was generated with mouse reference serum (Bethyl Laboratories, Inc., Montgomery, TX). Plates were read on an ELISA reader (Molecular Devices, Menlo Park, CA) and IgG calculated from the standard curve using Softmax software (Molecular Devices). To quantify anti-dsDNA Ig [36], 96-well plates were coated with 5 μg/ml dsDNA purified from calf thymus (Alpha Diagnostics, Inc. cat # DNAD25-N-1) in borate buffered saline. BALF, plasma samples or standards (mouse anti-dsDNA, Abcam clone HYB331/01) were loaded on the plate. Bound antibody was measured using horseradish peroxidase-labeled goat anti-mouse IgG (Fab2-specific) (Cappel Labs, cat # 3211021). Since this detector antibody reacts with all immunoglobulin classes (IgG, IgA, IgM, IgD and IgE), data were reported as anti-dsDNA Ig (ng/ml). Anti-dsDNA Ig in samples was determined from standard curve.

### Cytokine analyses

BALF supernatant and plasma were analyzed for the proinflammatory cytokines MCP-1, TNF-α, IFN-γ, IL-6, IL-1β, IL-10, IL-12p70, and IL-17a by flow cytometric bead array using Flex Set reagents (BD Biosciences, San Jose, CA). Sample data were acquired using a FACSCalibur flow cytometer (BD Biosciences) and cytokine concentrations were calculated from standard curves using FCAP Array Software (BD Biosciences). In addition to the Flex Set array, B cell stimulating cytokines in BALF supernatant and plasma were quantitated using mouse B-cell Activating Factor (BAFF) and Osteopontin DuoSets (R&D Systems, Minneapolis, MN) according to manufacturer’s instructions.

### Statistics

All treatments consisted of 8 mice per group and data presented as $\bar{x}$ ± SEM. A p value of < 0.05 was considered statistically different for all study outcomes. Grubb’s outlier test was performed and any identified outliers were excluded from statistical analysis. Data were analyzed using SigmaPlot 11.0 for Windows (Jandel Scientific; San Rafael, CA). Student’s t test was used to determine differences in CON-fed mice instilled with VEH or cSiO_2_. The effects of consuming DHA diets on cSiO_2_-triggered responses were analyzed by one-way analysis of variance (ANOVA) followed by Tukey’s test for multiple comparisons. If the assumption of normality or equal variance failed, Kruskal-Wallis one-way ANOVA on Ranks followed by Dunn’s test was used. The Spearman rank-order correlation coefficient was used to correlate DHA concentrations in diet to experimental endpoints in cSiO_2_-exposed NZBWF1 mice. Comparisons between VEH-exposed NZW/LacJ mice and cSiO_2_-exposed counterparts fed CON or DHA-enriched diets were conducted using Student’s t-test. If the assumption of normality or equal variance failed, differences were analyzed by Mann Whitney Rank Sum test.

## Results

### Consumption of DHA-rich microalgal oil dose-dependently increases tissue DHA

**Fig 2:**
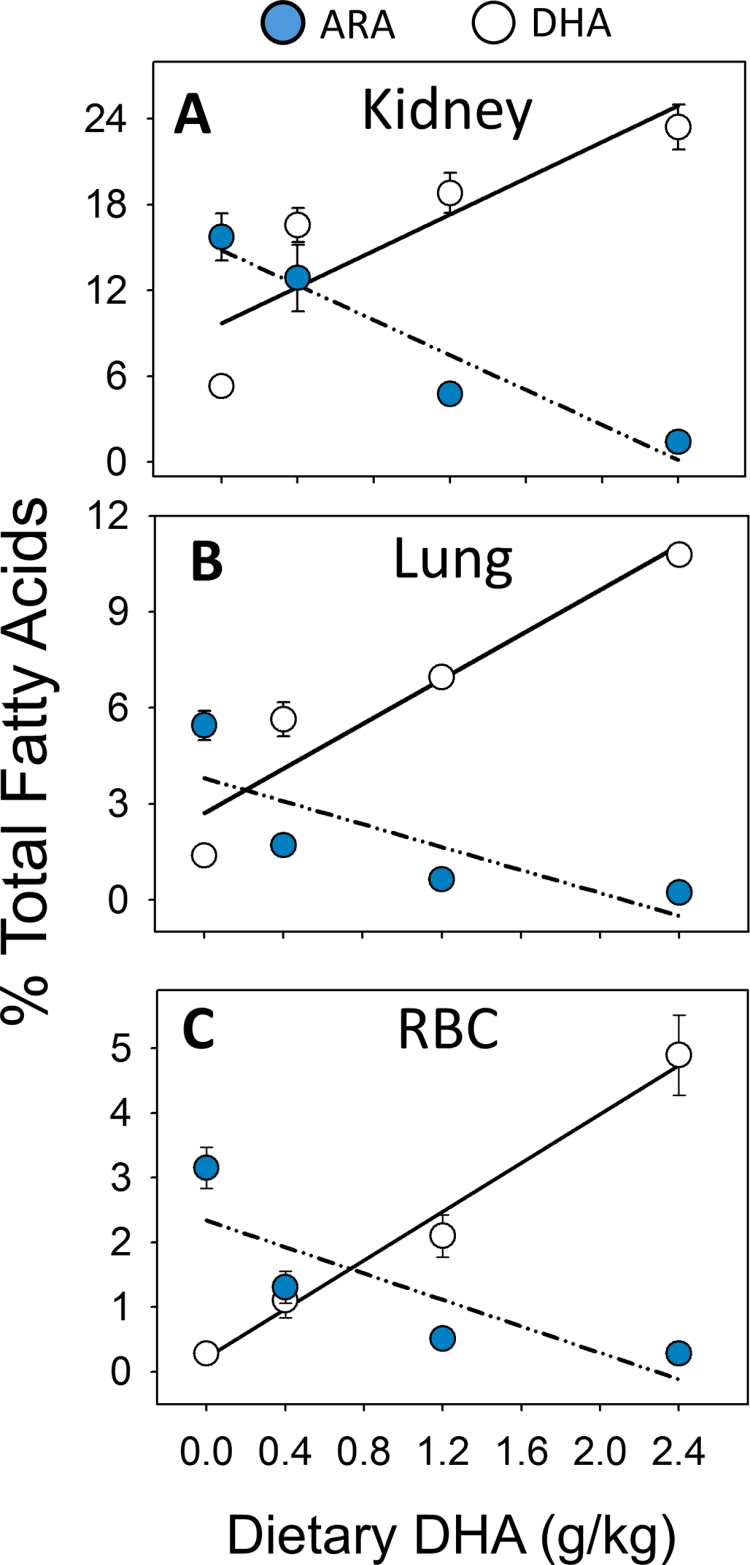
Consumption of microalgal oil dose-dependently increases DHA in kidney, lung and RBC. DHA incorporation in tissues increased in a dose-dependent manner that coincided with a reduction in ARA. (A) Kidney (r^2^ = 0.725, P<0.05) (B) lung (r^2^ = 0.961, p < 0.001), and (C) red blood cell (r^2^ = 0.876, p < 0.001).

Substitution of high oleic safflower oil in AIN-93G diets with increasing amounts of DHA oil (Tables 1 and 2) dose-dependently increased DHA content of lung (Fig 2A; S1 Table), red blood cells (Fig 2B; S2 Table) and kidney (Fig 2C; S3 Table) in NZBWF1 mice 12 wk after the last cSiO_2_ instillation_._ These effects co-occurred with reductions of the ω-6 PUFA ARA, suggesting that DHA effectively replaced ARA from cell membrane phospholipids in these three tissues. There was very little EPA detected in the tissues indicating that there was no retroconversion of DHA. Finally, cSiO_2_ exposure did not significantly affect DHA content in tissues of CON-fed mice.

### DHA consumption suppresses cSiO_2_-triggered proteinuria and lupus nephritis

**Fig 3:**
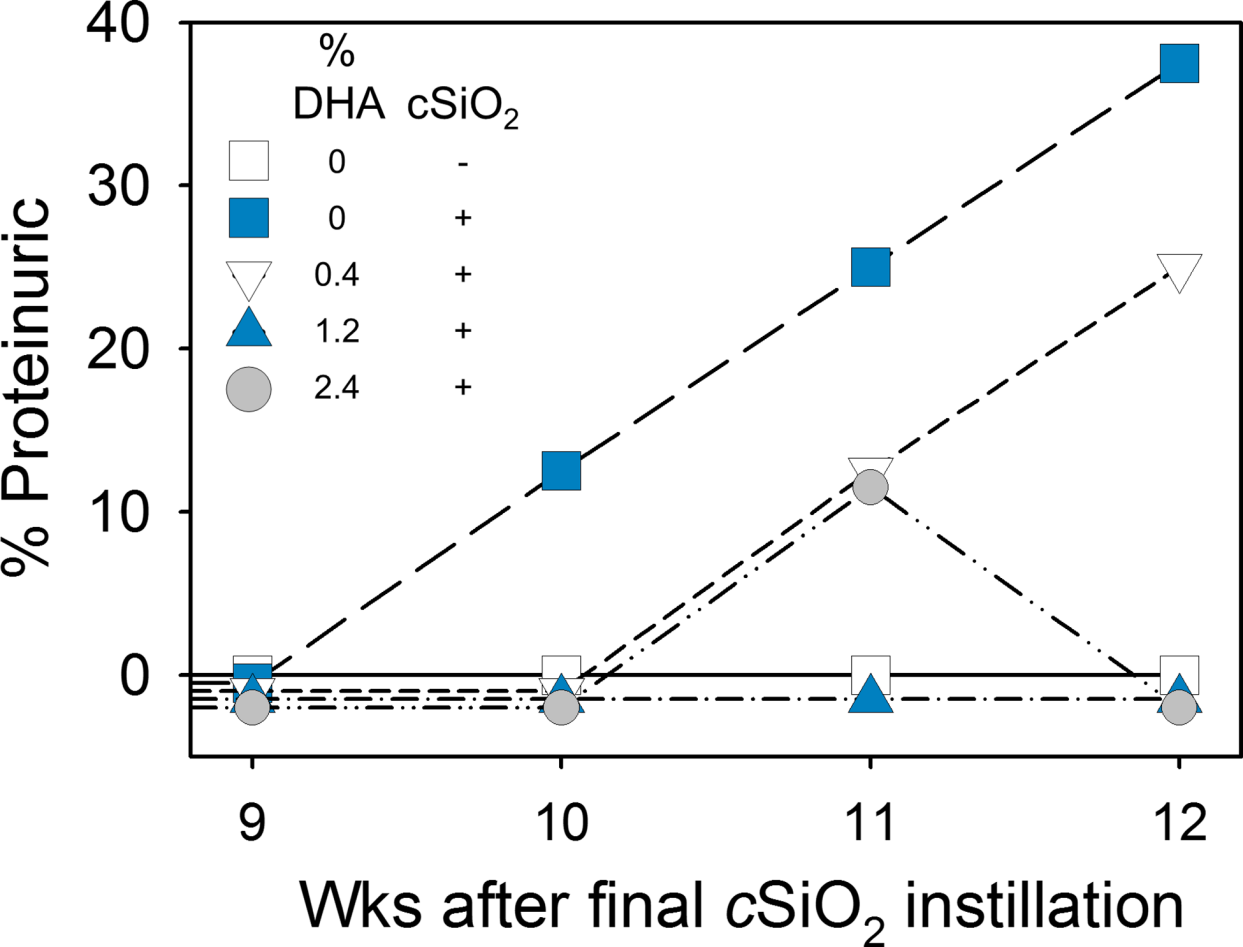
Dietary supplementation with DHA attenuates cSiO_2_-induced proteinuria in NZBWF1 mice. Proteinuria (>300 mg/dl) was monitored weekly until sacrifice after the final cSiO_2_ instillation. VEH-instilled NZBWF1 mice fed CON diet did not develop proteinuria. Proteinuria was undetectable in NZW/LacJ exposed to VEH or cSiO_2_ over the duration of the experiment.

Consistent with our prior findings [15], short-term repeated intranasal exposure to cSiO_2_ triggered the premature onset of proteinuria in CON-fed NZBWF1 mice as compared to VEH-instilled CON-fed mice (Fig 3). Proteinuria in cSiO_2_-exposed animals was apparent beginning at 10 wk post-instillation (PI) with 38% of this group responding by 12 wk PI. In contrast, cSiO_2_-exposed NZBWF1 mice fed 0.4% DHA showed delayed proteinuria onset, first detectable 11 wk PI; by 12 wk PI, 25% of this group was affected. None of the cSiO_2_-exposed NZBWF1 mice fed either 1.2% or 2.4% DHA exhibited proteinuria upon termination at 12 wk PI. Proteinuria was not evident at 12 wk PI in VEH- or cSiO_2_-instilled NZW/LacJ mice, which were controls without genetic susceptibility to autoimmunity.

**Fig 4:**
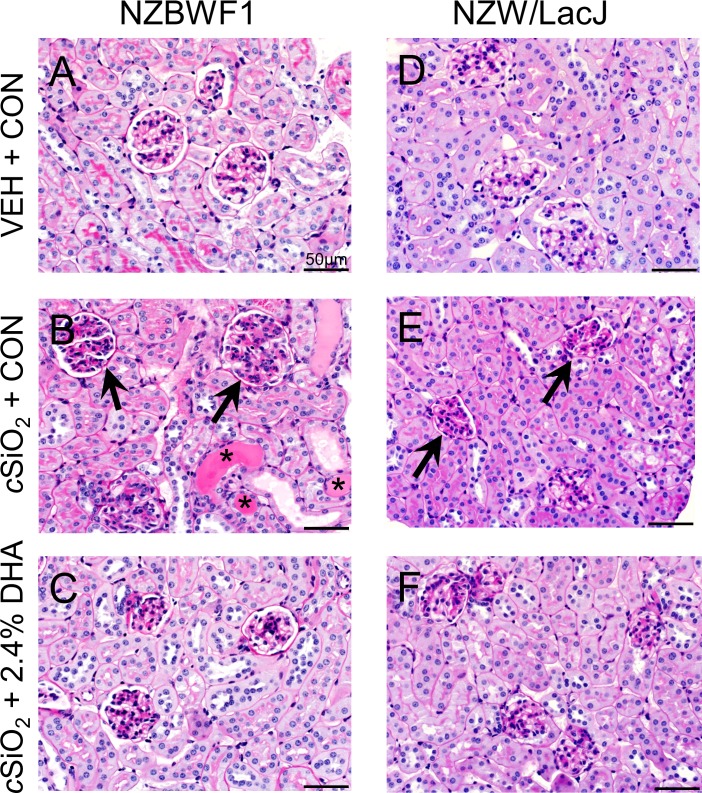
DHA consumption suppresses cSiO2-induced glomerulonephritis in NZBWF1 mice. Representative photomicrographs of H&E stained kidney section in NZBWF1 (A-C) and NZW/LacJ (D-F). Letters indicate CON-fed, VEH-exposed mice (A, D), CON-fed, cSiO2-exposed mice (B, E) and 2.4% DHA-fed, cSiO2-exposed mice(C, F). CON-fed NZBWF1 mice instilled with cSiO2 (B) developed extensive glomerulonephritis (black arrows) and tubular proteinosis (*). Mild histopathological lesions were also observed in some NZW/LacJ mice exposed to cSiO2 (E). Dietary supplementation with 2.4% DHA decreased severity of lesions in cSiO2-exposed NZBWF1(C), and NZW/LacJ mice (F).

**Fig 5:**
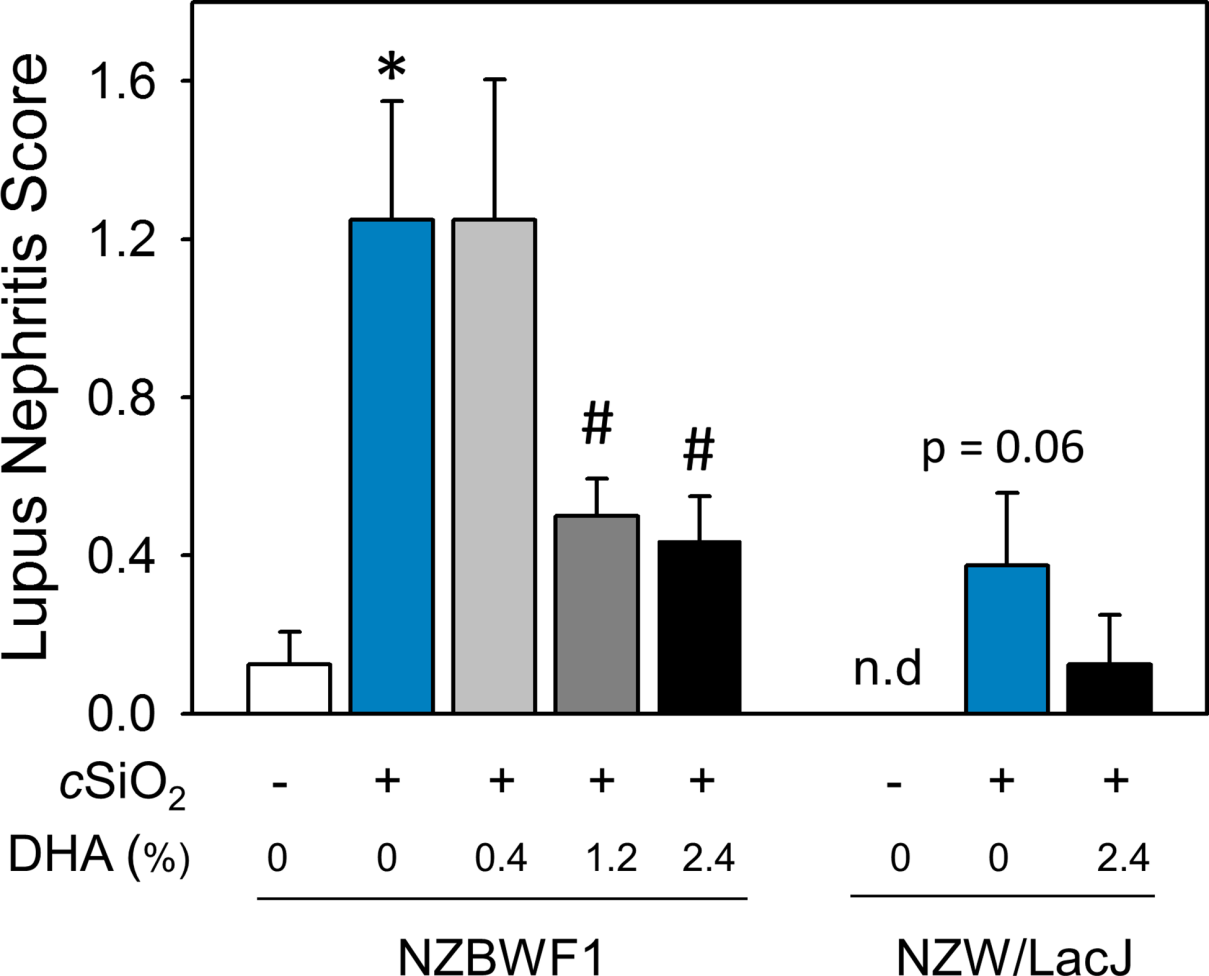
DHA dose-dependently reduces severity of lupus nephritis in cSiO2-exposed NZBWF1 mice. NZBWF1 and NZW/LacJ mice were individually graded following the modified ISN/RPS lupus nephritis classification system as described in Materials and Methods. Slide sections from kidneys were graded as follows: (0) no tubular proteinosis; (1) mild tubular proteinosis, early sclerosis, and mild crescent formation; (2) moderate tubular proteinosis, early sclerosis, and crescent formation; (3) marked tubular proteinosis with diffuse global proliferative and sclerosing glomerulonephritis. Data are $\bar{x}$ ± SEM (n = 8). Symbols: * indicates significant difference from CON-fed mice instilled with VEH (p < 0.05); # indicates significant difference from CON-fed mice instilled with cSiO_2_ (p < 0.05). DHA dose-dependently reduced cSiO_2_-triggered lupus nephritis in NZBWF1 mice (r^2^ = -0.414, p < 0.05).

DHA’s preventative effects on cSiO_2_-triggered renal injury in NZBW1 mice were confirmed histologically. CON-fed mice instilled with cSiO_2_ exhibited moderate to severe diffuse glomerular hypercellularity, mesangial matrix expansion, lymphocytic infiltration, and tubular proteinosis characteristic of glomerulonephritis as compared to CON diet-fed mice instilled with VEH (Fig 4A and 4B). In contrast, NZBWF1 mice fed diets containing 2.4% DHA exhibited marked reduction of such lesions (Fig 4C). Individual NZBWF1 mice were graded for severity of lupus nephritis (Fig 5). CON-fed mice that received cSiO_2_ showed significantly higher lupus nephritis scores than CON-fed mice instilled with VEH. In contrast, consumption of diets containing 1.2 or 2.4% DHA markedly reduced severity of cSiO_2_-induced nephritis. Consumption of diets containing 0.4% DHA did not affect cSiO_2_-induced nephritis. Hence, DHA consumption suppressed cSiO_2_-triggered kidney injury in the female NZBWF1 mouse.

cSiO_2_ instillation also induced renal lesions indicative of mild nephritis in NZW/LacJ control mice PI (Figs 4E and 5). These results indicate that intranasal exposure to cSiO_2_ per se might be sufficient to induce nephritis even in the absence of strong genetic predisposition to autoimmunity. Consistent with effects of DHA observed in cSiO_2_-exposed NZBWF1 mice, supplementation with 2.4% DHA appeared to reduce the severity of kidney lesions in NZW/LacJ mice exposed to cSiO_2_ (Figs 4F and 5).

### DHA consumption abrogates cSiO_2_-induced inflammatory cell response in lungs

**Fig 6:**
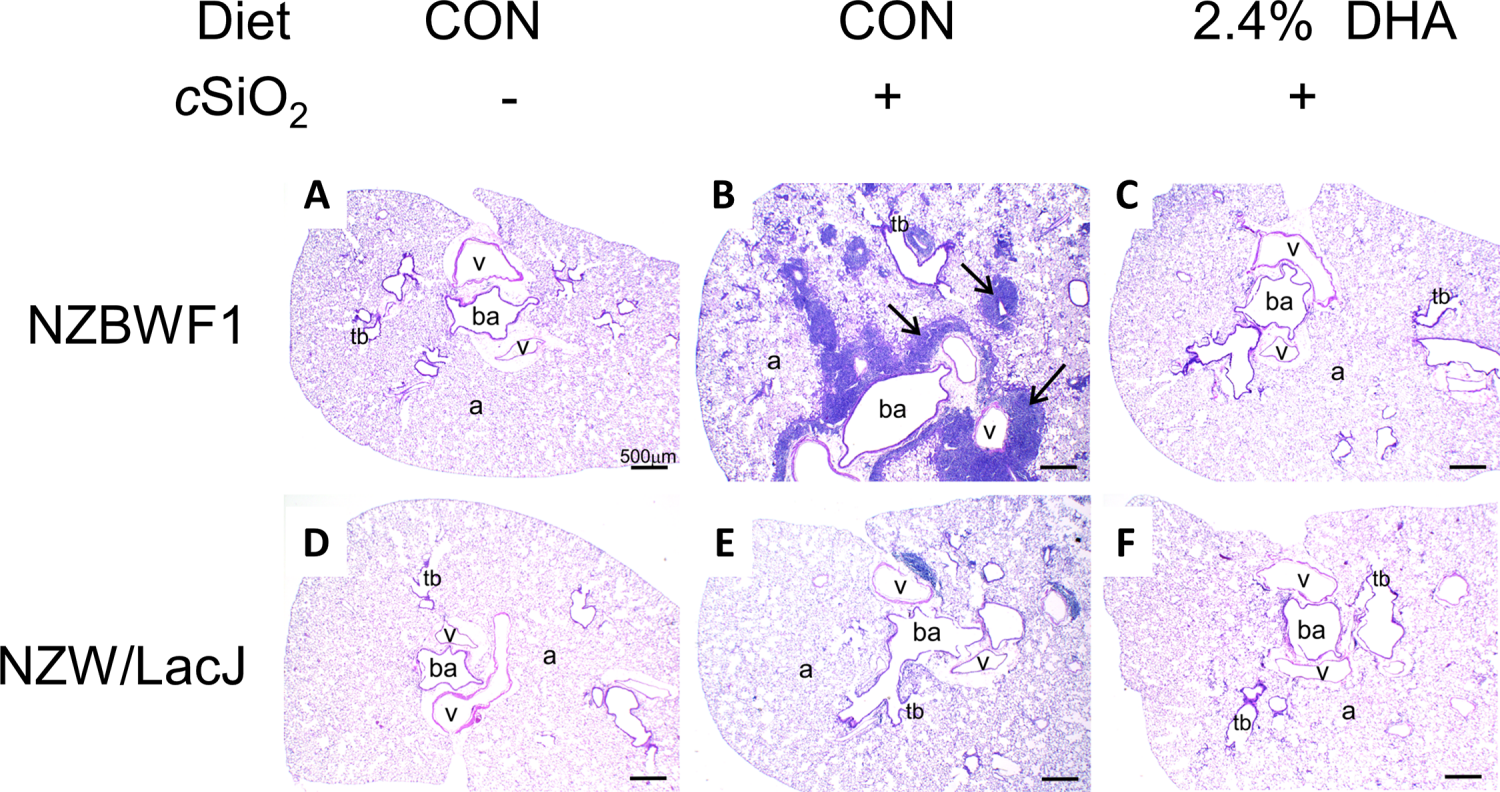
DHA supplementation prevents cSiO_2_-induced pneumonitis. Representative photomicrographs of H&E stained lung sections from NZBWF1 (A-C) and NZW/LacJ (D-F) mice exposed to VEH (A, D), cSiO_2_ fed CON diet (B, E), and cSiO_2_ fed 2.4% DHA (C, F). Black arrows in light photomicrographs denote marked leukocyte infiltration that circumvented both the vasculature and airways in the lung following cSiO_2_ exposure (B). Dietary DHA dramatically reduced cSiO_2_-induced pulmonary inflammation as evident by the absence of cellular accumulation in (C, F). Lymphocytic cell infiltration was semi-quantitatively graded as indicated in Table 3). Abbreviations: ba = bronchiolar airway, v = blood vessel, tb = terminal bronchiole, a = alveolus.

CON-fed NZBWF1 mice instilled with cSiO_2_ exhibited extensive inflammation in the lungs consisting of significant perivascular and peribronchiolar mononuclear cell infiltration compared to those instilled with VEH (Fig 6A and 6B). While diet supplemented with 0.4% DHA modestly reduced perivascular mononuclear infiltration, consumption of the 1.2 and 2.4% DHA diets dramatically suppressed cSiO_2_ induced mononuclear cell infiltration (Fig 6C). NZW/LacJ mice fed CON diet and intranasally instilled with cSiO_2_ also developed pulmonary mononuclear cell infiltration (Fig 6D and 6E), but this response was much less severe than in NZBWF1 mice. Nonetheless, consumption of 2.4% DHA reduced cSiO_2_-triggered mononuclear cell infiltration in this mouse strain (Fig 6F).

Observations in H&E stained serial lung sections from NZBWF1 and NZW/LacJ mice were confirmed by semi-quantitatively grading severity of pulmonary inflammation (Table 3). cSiO_2_ induced lymphocytic cell infiltration in both CON diet-fed NZBWF1 and NZW/LacJ mice; however, the response induced in autoimmune-prone NZBWF1 mice was considerably more severe. DHA diets were effective at reducing this infiltration in NZBWF1 mice in a dose-dependent manner. Infiltration of mononuclear cells in cSiO_2_-treated NZW/LacJ mice was also suppressed by feeding diets containing 2.4% DHA. Dietary supplementation with 2.4% DHA reduced alveolitis in NZBWF1 mice as compared to CON diet-fed mice, whereas consumption of 0.4 and 1.2% DHA diets had no effect. These effects were not observed in NZW/LacJ mice. Finally, DHA consumption did not influence cSiO_2_-triggered alveolar proteinosis in NZBWF1 or NZW/LacJ mice. Consequently, DHA supplementation primarily targeted lymphocytic cell infiltration in the lung.

**Table 3:** Histopathological assessment of cSiO_2_-triggered pulmonary lung inflammation in lupus-prone NZBWF1 and NZW/LacJ mice fed DHA.

			Histopathologic Severity Grade		
Strain	cSiO_2_	Diet	Lymphocytic cell infiltration	Alveolitis	Alveolar proteinosis
NZBWF1	-	CON	0.8 ± 0.2	0.0 ± 0.0	0.0 ± 0.0
	+	CON	3.8 ± 0.2*	2.9 ± 0.1*	2.5 ± 0.2*
	+	0.4% DHA	2.9 ± 0.3	2.6 ± 0.2	2.6 ± 0.2
	+	1.2% DHA	1.4 ± 0.2^#^	2.3 ± 0.2	2.5 ± 0.2
	+	2.4% DHA	1.3 ± 0.2^#^	1.9 ± 0.2^#^	2.5 ± 0.2
NZW/LacJ	-	CON	0.0 ± 0.0	0.0 ± 0.0	0.0 ± 0.0
	+	CON	1.8 ± 0.1*	2.3 ± 0.3*	2.3 ± 0.3*
	+	2.4% DHA	1.0 ± 0.0^#^	2.1 ± 0.4	2.1 ± 0.4

Mice were graded individually for severity of lung inflammation (% of total pulmonary tissue examined) as follows: 0, no changes; 1, minimal (<10%); 2, slight (10–25%); 3, moderate (26–50%); 4, severe (51–75%) 5; very severe (>75%) of total area affected. Data are mean ± SEM (n = 8/gp). Symbols

* indicates significant difference from CON-fed mice instilled with VEH (p < 0.05)

# indicates significant difference from CON-fed mice instilled with cSiO2 (p < 0.05). DHA dose-dependently decreased lymphocytic cell infiltration (r2 = -0.845, p < 0.001) and alveolitis (r2 = -0.622, p < 0.001) in NZBWF1 mice.

**Fig 7:**
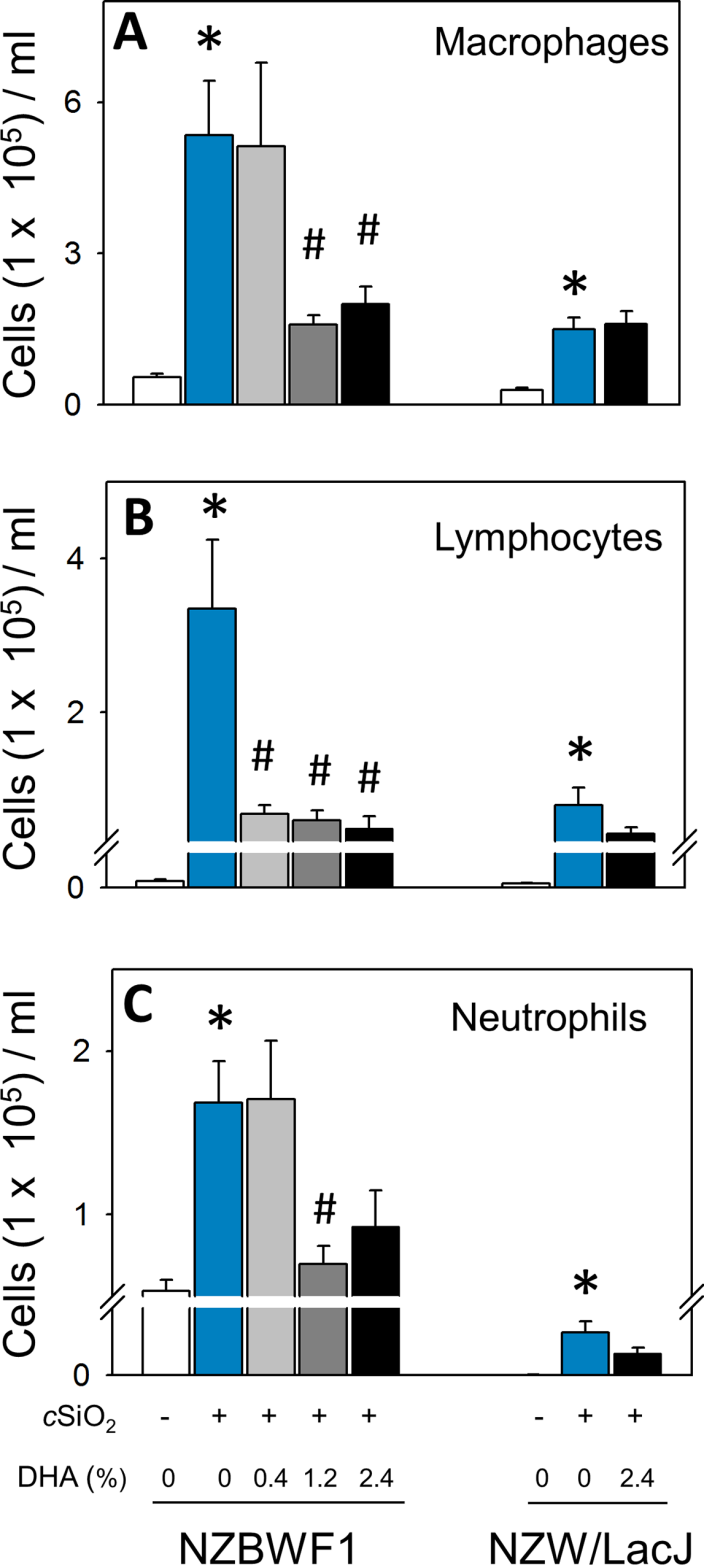
DHA consumption abrogates cSiO_2_-induced macrophage, lymphocyte, and polymorphonuclear leukocyte accumulation in BALF. Differential counts of macrophages (A), lymphocytes (B), and neutrophils (C) in BALF of NZBWF1 and NZW/LacJ mice. Data are $\bar{x}$ ± SEM (n = 8). Symbols: * indicates significant difference from CON-fed mice instilled with VEH (p < 0.05); # indicates significant difference from CON-fed mice instilled with cSiO_2_ (p < 0.05). DHA dose-dependently decreased macrophages (r^2^ = -0.545, p < 0.05), lymphocytes (r^2^ = -0.599, p < 0.001), and neutrophils (r^2^ = -0.448, p < 0.05) in NZBWF1 mice.

Macrophages, lymphocytes, and neutrophils were dramatically increased in the BALF of CON-fed NZBWF1 mice instilled with cSiO_2_, while NZW/LacJ mice exhibited slighter increases (Fig 7A–7C). Total cell counts in BALF of cSiO_2_-exposed NZBWF1 mice were approximately 3-fold higher relative to NZW/LacJ mice. Consumption of 1.2 and 2.4% DHA markedly impaired macrophage, lymphocyte, and neutrophil cellular infiltration in cSiO_2_-exposed NZBWF1 mice. Consumption of the 0.4% DHA diet reduced lymphocyte counts in BALF but did not affect macrophage or neutrophil infiltration. cSiO_2_-exposed NZW/LacJ mice fed 2.4% DHA also showed a trend towards decreased lymphocyte and neutrophil infiltration compared to CON-fed mice.

### Dietary DHA prevents cSiO_2_-induced ELT neogenesis and autoimmunity in the lung

**Fig 8:**
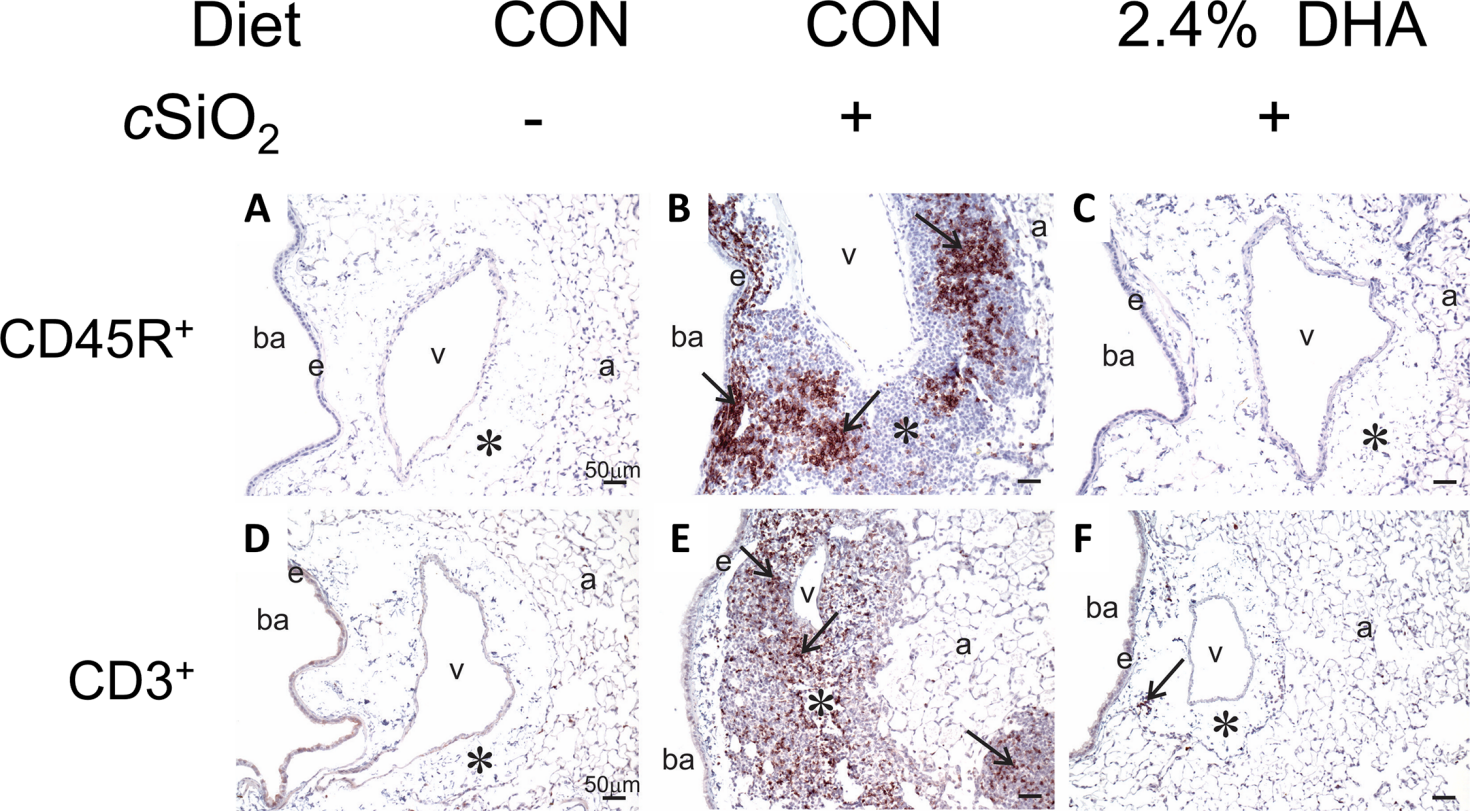
B and T cell infiltration in lungs of NZBWF1 mice following cSiO_2_ exposure is prevented by dietary supplementation with DHA. Representative light photomicrographs of lung tissue sections from CON-fed NZBWF1 mice treated with VEH (A, D), CON-fed NZBWF1 mice treated with cSiO_2_ (B, E), 2.4% DHA-fed mice treated with cSiO_2_ (C, F). Lung sections were stained with either CD45R to identify B-lymphocytes (A-C) or CD3 to identify T cells (D-F) and counterstained with hematoxylin. Inflammatory cell infiltrates in peribronchiolar and perivascular interstitium induced by cSiO_2_ (asterisk in B and E) consisted of both B and T lymphocytes as indicated by positive immunohistochemical staining (black arrows in B and E, respectively). B-lymphocytes tended to form distinct aggregates whereas T lymphocytes, which were more diffusely, scattered throughout lymphoid cells aggregates. Dietary DHA blocked B and T cell accumulation in lungs of cSiO_2_-treated NZBWF1 mice as evident by marked reduction in CD45R^+^ and CD3^+^ cells. Abbreviations: ba = bronchiolar airway, e = airway epithelium, a = alveolus, v = blood vessel, * = interstitium.

**Fig 9:**
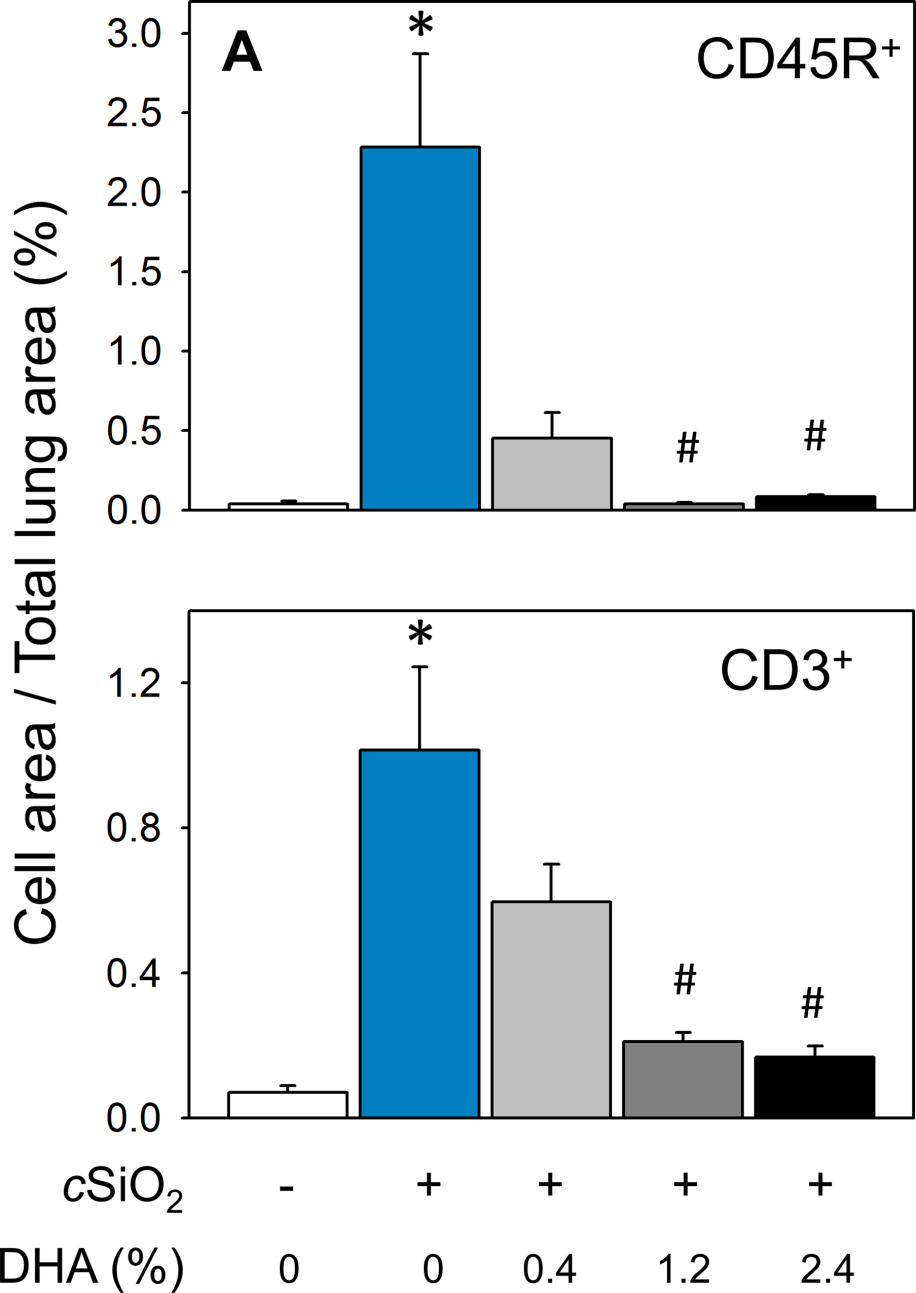
cSiO_2_-triggered B and T cell infiltration in lungs of NZBWF1 mice is dose-dependently prevented by DHA consumption. Morphometric quantitation of B cell (A) and T cell (B) cellular infiltration in lung parenchyma in CON- and DHA-fed mice exposed to VEH or cSiO_2_. Data are $\bar{x}$ ± SEM (n = 8). Symbols: * indicates significant difference from CON-fed mice instilled with VEH (p < 0.05); # indicates significant difference from CON-fed mice instilled with cSiO_2_ (p < 0.05). DHA consumption dose-dependently decreased CD45R^+^ (r^2^ = -0.707, p<0.001) and CD3^+^ (r^2^ = -0.728, p<0.001) cellular infiltration.

Lymphocyte populations in lungs of NZBWF1 mice were characterized immunohistochemically employing antibodies to the pan B and T lymphocyte markers CD45R and CD3, respectively. Both CD45R^+^ (Fig 8A and 8B) and CD3^+^ cells (Fig 8D and 8E) indicative of cSiO_2_-induced ELT neogenesis were detected in lung parenchyma in CON-fed mice. Morphometry revealed that CD45R^+^ cells (Fig 9A) were more abundant in lung parenchyma than CD3^+^ cells (Fig 9B). These findings confirmed previous observations that intranasal cSiO_2_ exposure induces ELT development in NZBWF1 mice [15]. Dietary supplementation with 0.4, 1.2, and 2.4% DHA dose-dependently suppressed cSiO_2_-triggered B and T cell expansion (Figs 8C, 8F and 9). The CD45R^+^ cell area in lung parenchyma was reduced by DHA consumption 80, 98, and 96%, respectively, relative to cSiO_2_-exposed NZBWF1 fed CON diet (Fig 9A). In analogous fashion, DHA supplementation reduced CD3^+^ cell area in lung parenchyma by 41, 79, and 83%, respectively (Fig 9B).

**Fig 10:**
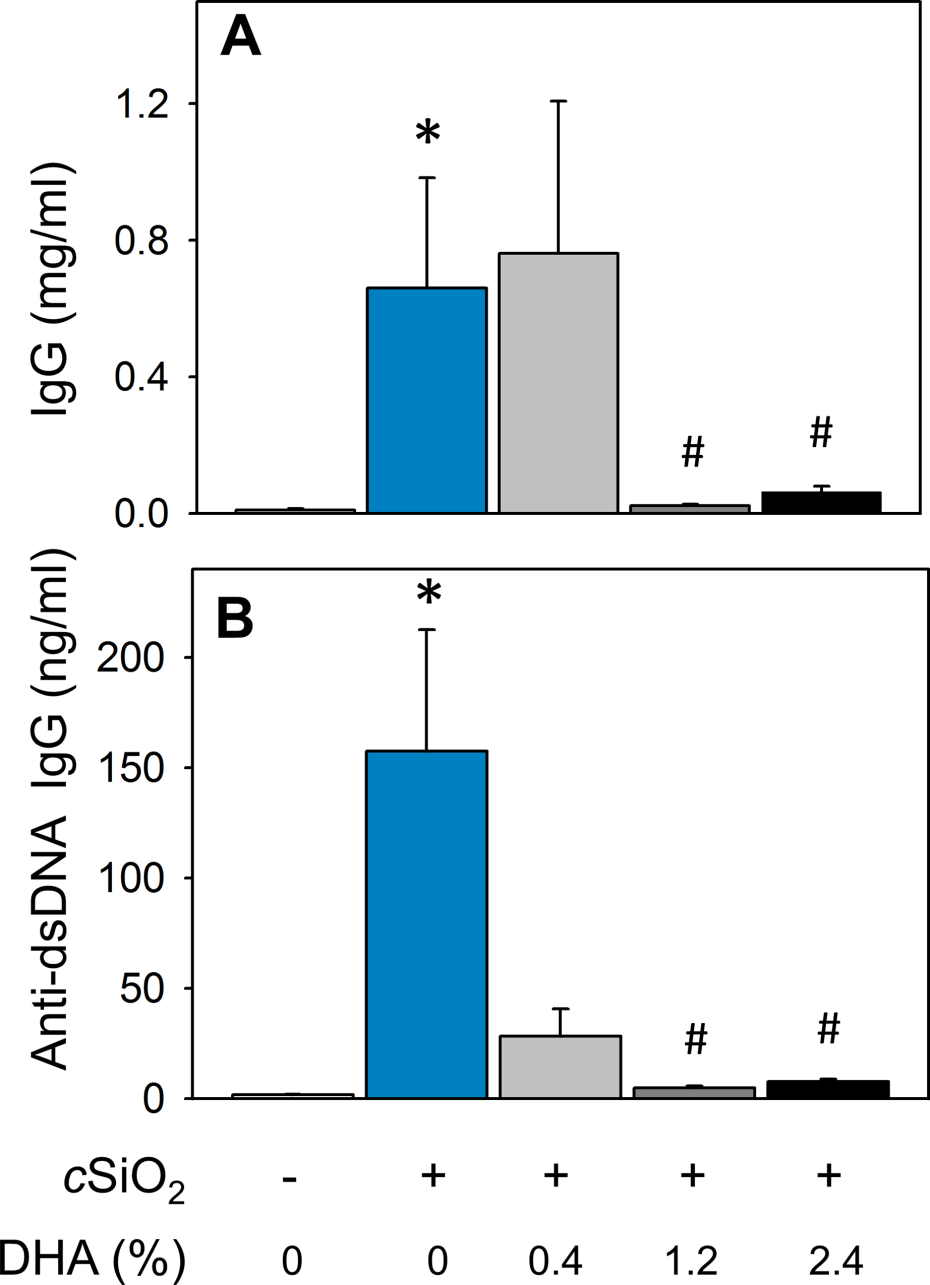
Dietary DHA suppresses cSiO_2_ -induced elevation of total IgG and anti-dsDNA Ig in BALF of NZBWF1 mice. Total IgG (A) and anti-dsDNA Ig (B) in BALF of NZBWF1 mice was quantitated by ELISA. Data are $\bar{x}$ ± SEM (n = 8). Symbols: * indicates significant difference from CON-fed mice instilled with VEH (p < 0.05); # indicates significant difference from CON-fed mice instilled with cSiO_2_ (p < 0.05). Dietary DHA dose-dependently decreased total IgG (r^2^ = -0.574, p < 0.001) and anti-dsDNA Ig (r^2^ = -0.546, p < 0.05) in BALF.

Consistent with immunohistochemical findings, cSiO_2_ triggered robust elevations in total IgG and anti-dsDNA Ig concentrations, clinical signs of active lupus, in the BALF of CON diet-fed NZBWF1 mice (Fig 10A and 10B). cSiO_2_-triggered elevation in total IgG was suppressed in mice fed diets containing 1.2 and 2.4% DHA. Likewise, DHA feeding at 0.4, 1.2 and 2.4% decreased anti-dsDNA Ig in BALF by 82, 97, and 95% respectively, relative to CON-fed cSiO_2_-exposed NZBWF. Accordingly, dietary DHA consumption dramatically diminished cSiO_2_-induced ELT neogenesis and production of total and autoreactive IgG production in the lung.

### DHA consumption suppresses cSiO_2_–triggered secretion of proinflammatory and B-cell stimulatory cytokines into BALF

**Fig 11:**
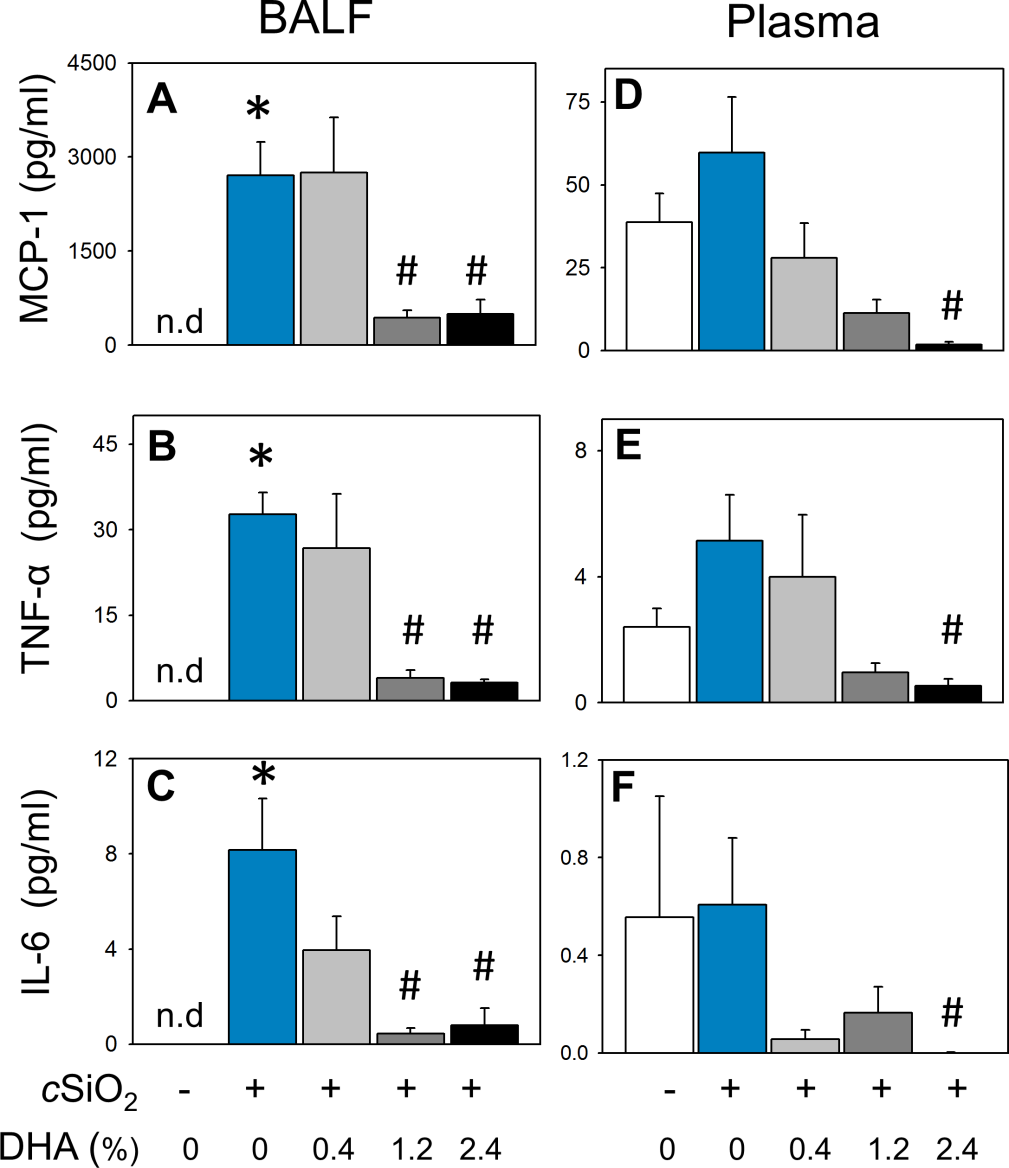
cSiO_2_ -induced elevations of proinflammatory cytokines MCP-1, TNF-α and IL-6 in BALF and plasma are decreased by DHA consumption in NZBWF1 mice. MCP-1 (A, D), TNF-α, (B, E) and IL-6 (C, F)) were quantitated in BALF (left panel) and plasma (right panel) by flow cytometric bead array. Data are $\bar{x}$ ± SEM (n = 8). The designation n.d. indicates below the limit of detection. Bars without same letter are significantly different (p<0.05). Symbols: * indicates significant difference from CON-fed mice instilled with VEH (p < 0.05); # indicates significant difference from CON-fed mice instilled with cSiO_2_ (p < 0.05). DHA dose-dependently decreased BALF concentrations of MCP-1 (r^2^ = -0.791, p < 0.001), TNF- α (r^2^ = -0.577 p < 0.001), and IL-6 (r^2^ = -0.810, p < 0.001). DHA dose-dependently decreased plasma MCP-1 (r^2^ = -0.871, p < 0.001) and TNF-α (r^2^ = -0.527, p < 0.05).

**Fig 12:**
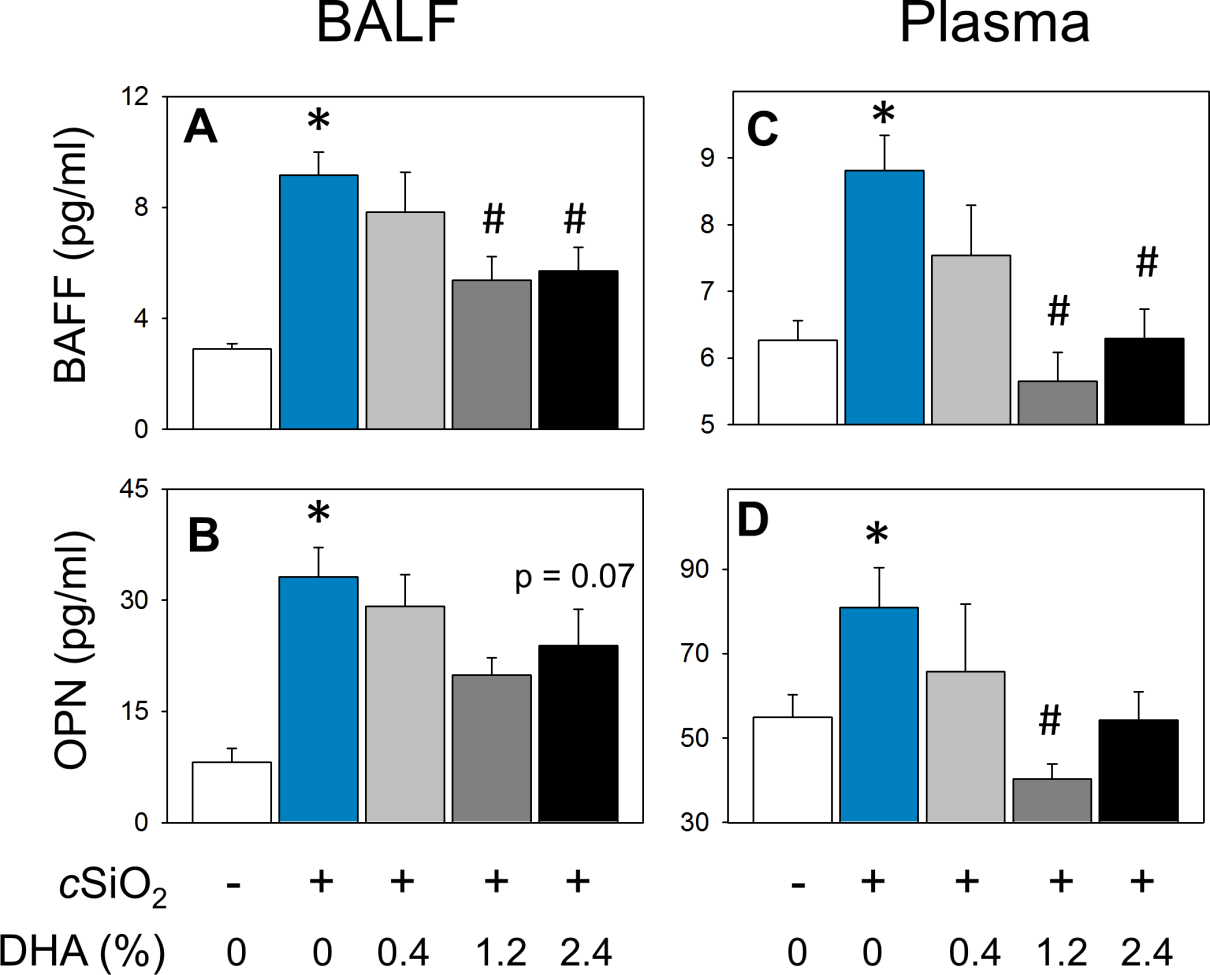
cSiO_2_ -induced elevation of B cell stimulating cytokines BAFF and osteopontin are decreased in BALF and plasma in NZBWF1 mice fed DHA. B cell stimulating cytokines B cell activating factor (BAFF) (A, C) and osteopontin (OPN) (B, D) were quantitated by ELISA in BALF (left panel) and plasma (right panel). Data are $\bar{x}$ ± SEM (n = 8). Symbols: * indicates significant difference from CON-fed mice instilled with VEH (p < 0.05); # indicates significant difference from CON-fed mice instilled with cSiO_2_ (p < 0.05). DHA dose-dependently decreased BAFF in BALF (r^2^ = -0.507, p < 0.05) and plasma (r^2^ = -0.539, p < 0.05). DHA dose-dependently decreased OPN in BALF (r^2^ = -0.330, p = 0.06) and in plasma (r^2^ = -0.493, p < 0.05).

cSiO_2_ instillation of CON-fed NZBWF1 mice elicited increased concentrations of MCP-1, TNF-α, and IL-6 in BALF compared to CON-fed mice instilled with VEH (Fig 11A–11C). DHA consumption dose-dependently attenuated these responses, with the 1.2 and 2.4% DHA diets being more efficacious than the 0.4% DHA diet. Other cytokines in the flow cytometric bead panel IFN-γ, IL-1β, IL-10, IL-12p70, and IL-17a fell below the limit of detection. The effects of DHA on two B-cell stimulating cytokines, BAFF and OPN, were also assessed in BALF of NZBWF1 mice. Both cytokines were significantly induced in CON-fed mice instilled with cSiO_2_ (Fig 12A and 12B). Dietary supplementation with DHA dose-dependently decreased *c*iSO_2_-induced BAFF and a similar trend was observed for *c*iSO_2_-induced OPN.

### Dietary DHA attenuates cSiO_2_-induced systemic autoantibody and cytokine elevation

**Fig 13:**
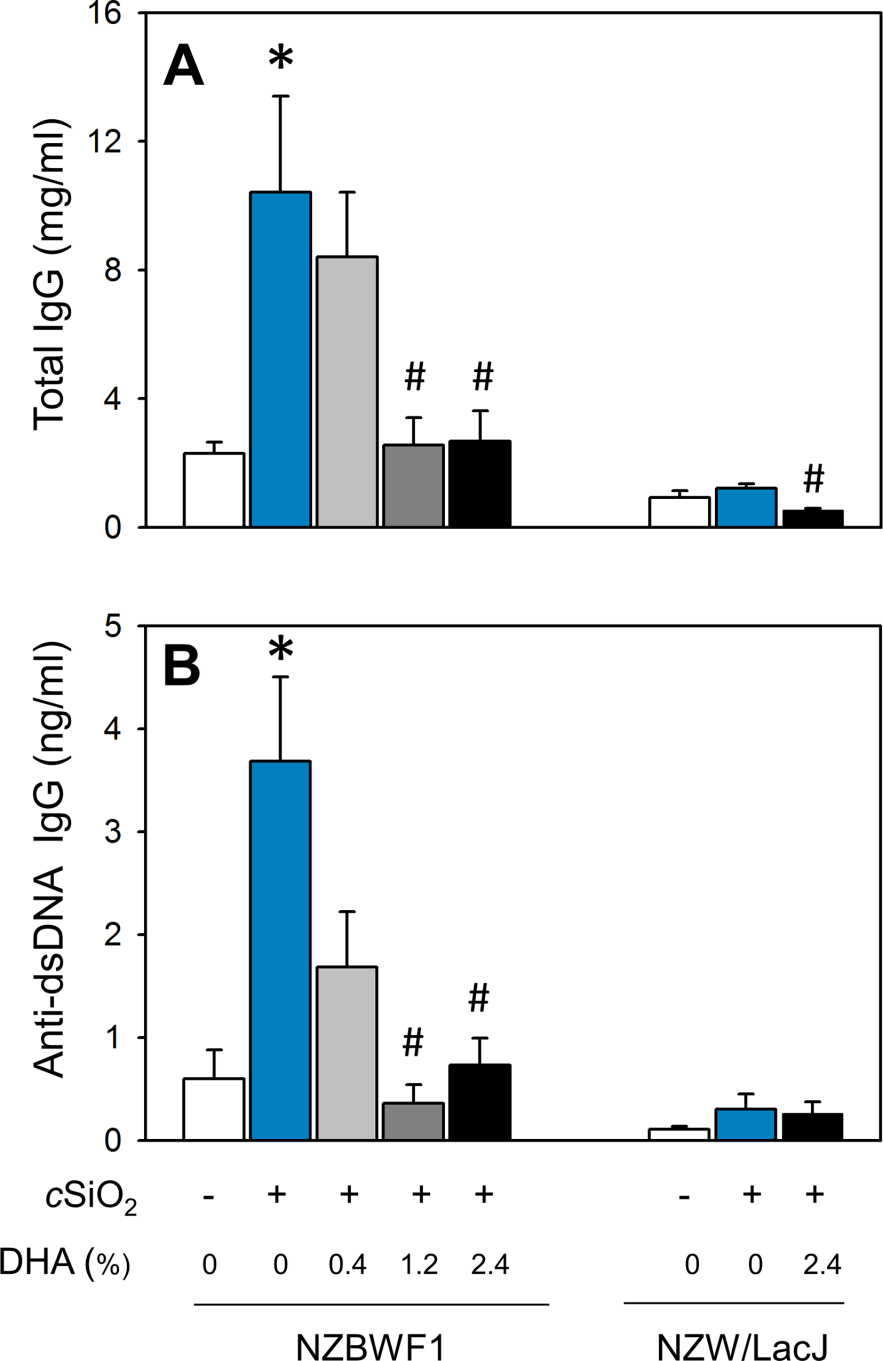
Dietary DHA attenuates cSiO_2_ -induced elevation of total IgG and anti-dsDNA Ig in plasma of NZBWF1 and NZW/LacJ mice. Total IgG (A) and anti-dsDNA Ig (B) in plasma of NZBWF1 mice was quantitated by ELISA. Data are $\bar{x}$ ± SEM (n = 8). Symbols: * indicates significant difference from CON-fed mice instilled with VEH (p < 0.05); # indicates significant difference from CON-fed mice instilled with cSiO_2_ (p < 0.05). DHA dose-dependently decreased plasma total IgG in NZBWF1 (r^2^ = -0.493, p < 0.05) and NZW/LacJ mice (r^2^ = -0.814, p = < 0.001). DHA dose-dependently decreased plasma anti-dsDNA Ig in NZBWF1 (r^2^ = -0.567, p < 0.001).

As observed in BALF, CON-fed NZBWF1 mice instilled with cSiO_2_ exhibited plasma elevations of total IgG and anti-dsDNA Ig relative to their VEH-exposed counterparts (Fig 13A and 13B). Consumption of diets containing 1.2 and 2.4% DHA suppressed cSiO_2_-induced IgG elevation. Dietary DHA also dose-dependently decreased anti-dsDNA Ig concentrations in plasma of NZBWF1 mice. Differences in total IgG were not evident between CON-fed NZW/LacJ mice exposed to VEH or cSiO_2_. Interestingly, cSiO_2_-exposed NZW/LacJ mice fed DHA had significantly decreased total IgG in plasma compared to cSiO_2_-exposed mice fed CON diet (Fig 13A). Differences in anti-dsDNA Ig were not detected among any NZW/LacJ treatment groups (Fig 13B).

Plasma was also analyzed for cytokines to evaluate systemic effects of cSiO_2_ and DHA. In CON-fed NZBWF1 mice, cSiO_2_ instillation caused an elevation in systemic MCP-1 and TNF-α. Dose-dependent reductions in MCP-1 and TNF-in plasma occurred because of dietary treatment with DHA (Fig 11D and 11E). cSiO_2_ treatment did not increase IL-6 in plasma but there was a trend toward decreased levels of this cytokine in DHA-fed mice (Fig 11F). Other cytokines IFN-γ, IL-1β, IL-10, IL-12p70, and IL-17a fell below the limit of detection. cSiO_2_ exposure also induced plasma BAFF in CON-fed NZBWF1 mice (Fig 12C). Consumption of 1.2, and 2.4% DHA diets significantly reduced cSiO_2_-induced systemic BAFF responses. Similar trends were observed for OPN with 1.2% DHA diet significantly suppressing this B cell factor (Fig 12D).

## Discussion

Heredity is widely viewed to be critical in the manifestation of autoimmunity in an individual; however, the exposome can impact both latency and severity of an AD [37]. This investigation is unique because it is the first to employ an animal model genetically predisposed to lupus, a prototypical AD, to identify how two highly relevant and countervailing exposome elements, cSiO_2_ exposure, and ω-3 PUFA content of the diet, influence latency, and progression of autoimmunity. Importantly, we demonstrate that dietary DHA dose-dependently inhibited cSiO_2_-triggered inflammation, ELT neogenesis and autoantibody production in the lung. Moreover, these effects were recapitulated systemically as evidenced by concurrent DHA-mediated suppression of cSiO_2_-induced elevations in cytokines and autoantibodies in plasma. Lastly, DHA consumption acted further downstream at the kidney by preventing glomerulonephritis and resultant proteinuria. Collectively, these data support the contention that triggering of autoimmunity by cSiO_2_ might be prevented or delayed by modulating dietary lipid composition.

Our findings confirm recent observations that weekly intranasal exposure of female NZBWF1 mice to 1 mg cSiO_2_ for 4 wk triggers autoimmune responses in the lung, blood and kidney [15]. Notably, there was robust development of ELT in the lung as evidenced by extensive perivascular and peribronchial lymphoplasmacytic infiltration consisting of T cells, B cells, and IgG-producing plasma cells. We further detected elevated concentrations of autoantibodies, cytokines (TNF-α and IL-6), the chemokine MCP-1 and B cell stimulatory factors (BAFF, OPN) in BALF and plasma. Both reduced latency and increased intensity of glomerulonephritis paralleled inflammatory events in the lung and plasma. Hence, our current and previous findings strongly suggest that, following airway exposure to cSiO_2_, the lung might serve as a staging point for triggering systemic autoimmunity and lupus nephritis.

**Fig 14:**
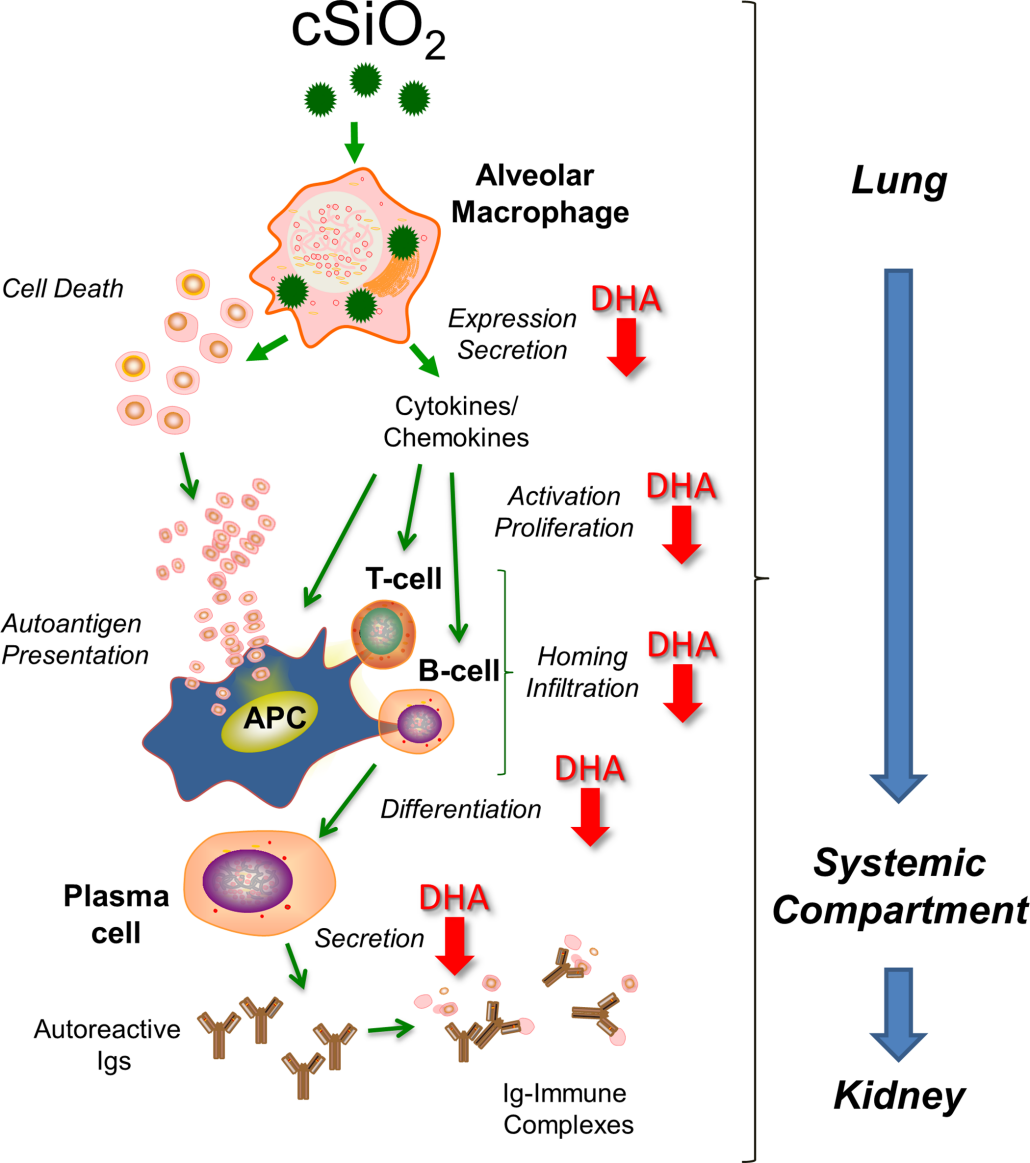
Putative mechanisms for DHA-mediated suppression of cSiO_2_-induced autoimmunity. The data presented here suggest that cSiO_2_-triggered pulmonary inflammation and ectopic lymphoid neogenesis drive systemic autoimmunity and glomerulonephritis in the female NZBWF1 mouse. Red downward arrows indicate potential action sites for suppressive effects of DHA that can be further predicted from these data.

Initiation of lupus involves, in part, impaired phagocytosis of dead or dying cells leading to aberrant autoantigen presentation and production of autoantibodies, an immunological hallmark for this AD [38–43]. These autoantibodies form complexes with self-antigens (e.g. dsDNA and nucleosome fractions) that subsequently deposit widely in tissues. In the kidney, such immune complexes promote proinflammatory cytokine and chemokine production that mediate infiltration by mononuclear cells and consequent tissue injury [44–47]. Lupus morbidity and mortality often correspond with severity of autoimmune glomerulonephritis [48]. It has been convincingly established that airway exposure to cSiO_2_ or other particles unleashes a vicious, repetitive cycle in alveolar macrophages of phagocytosis → lysosomal membrane permeabilization → inflammasome activation → cell death → release of free cSiO_2_ particles → phagocytosis that drives chronic inflammation in the lung [49, 50]. This cycle may deplete the alveolar macrophage population, thereby promoting cell debris accumulation, secondary necrosis, alarmin release, and self-antigen presentation that culminate in early loss of tolerance with consequent production of pathogenic autoantibodies. Intriguingly, many of the processes that drive toxicity of cSiO_2_ particles in the lung overlap with well-established early events in lupus pathogenesis [51]. Consistent with this model, our findings, summarized in Fig 14, suggest that DHA consumption attenuates cSiO2-induced i) cytokine and chemokine release, ii)lymphocyte activation, proliferation and homing, iii) B cell differentiation to plasma cells and iv)IgG secretion by plasma cells.

Several points are noteworthy regarding the cSiO_2_ dosing regimen employed here. Because the mouse lifetime is approximately 30 to 40 times less than that of a person, a contracted period of cSiO_2_ dosing (4 wk) was used to mimic long-term chronic human exposure. The total cSiO_2_ dose in this study approximates one half of a human lifetime occupational exposure at the current OSHA exposure limit [3]. Although other routes of cSiO_2_ administration in mice have been reported such as intratracheal and trans-oral instillation (oropharyngeal aspiration), we chose intranasal instillation based on its successful prior use in NZBWF1 and NZM2410 mice [12, 13, 52, 15]. In further support of our choice, an investigation comparing these approaches reported in mice receiving a single 1 mg dose of cSiO_2_ by each technique elicited similarly robust pulmonary inflammation [53].

Little is known regarding the distribution of cSiO_2_ particles outside of the lung following exposure and how such distribution might impact chronic toxicity to cSiO_2_. Sparse case reports from individuals occupationally exposed to cSiO_2_ have reported not only the development of typical silica-induced pulmonary diseases (i.e. silicosis), but also observations of cSiO_2_ particles in peripheral organs such the lymph node, spleen, and kidney [54]. However, computational models have predicted that distribution of cSiO_2_ particles to these compartments is negligible[55], suggesting that pathological changes observed in these distal tissues are more likely a result of immune system dysfunction rather than direct effects of particle toxicity. An alternative possibility for the pronounced suppressive effect of DHA we observed in both the lung and distal organs of cSiO_2_-exposed NZBWF1 mice is dependent on restoration of the function of the mucociliary escalator, which mediates clearance of particle-laden alveolar macrophages from the respiratory tract. In a process termed "particle overload", high particle lung burdens (such as those encountered by chronic exposure in occupational settings and doses of cSiO_2_ utilized in this study), result in alveolar macrophages saturated with engulfed cSiO_2_ that cease translocation to the mucociliary escalator, culminating in inhibition of this clearance mechanism and contributing to chronic toxicity [56, 57]. It is tempting to speculate that DHA could facilitate enhanced phagocytosis of cSiO_2_ particles, possibly by increasing the fluidity of macrophage cellular membranes [58], which may facilitate pulmonary clearance of cSiO_2_-laden alveolar macrophages in lieu of the high concentrations of cSiO_2_. In support of this possibility, previous studies have demonstrated that DHA and/or its metabolites enhanced phagocytosis of zymosan beads [59, 60]; however no studies have specifically addressed their effect on phagocytosis or clearance of environmental particulates.

The NZW/LacJ strain was selected as a control for this study because it is a parental strain for NZBWF1 that does not spontaneously develop glomerulonephritis [10, 26]. It was therefore notable that NZW/LacJ mice also exhibited modest pneumonitis and nephritis after cSiO_2_ exposure and that this could be prevented by consumption of DHA. Interestingly, we similarly found that cSiO_2_ induces similar effects in C57Bl6 mice, which are also not prone to autoimmunity [15].

Numerous preclinical/clinical studies have demonstrated that dietary ω-3 PUFA supplementation suppresses and sometimes even reverses innate immune cell-driven inflammation thus making these dietary lipids attractive candidates for the prevention/treatment of chronic inflammatory diseases [21]. For example, ω-3 PUFAs suppress proinflammatory cytokine production, lymphocyte proliferation, cytotoxic T cell activity, natural killer cell activity, macrophage-mediated cytotoxicity, neutrophil/monocyte chemotaxis, MHCII expression and antigen presentation. Mechanistically, the anti-inflammatory effects of ω-3 PUFAs have been linked to alterations in: 1) production of bioactive lipid mediators, 2) intracellular signaling, transcription factor activity, and gene expression; and 3) membrane structure/function [61].

The potential of fish oil or semi-purified ω-3 PUFAs to specifically delay, prevent, and ameliorate autoimmunity has been investigated extensively in lupus-prone mouse models. Early studies with NZBWF1 mice demonstrated that initiating feeding of menhaden oil, DHA ethyl ester or EPA ethyl ester early in life markedly reduce severity and incidence of renal disease as well as extend the lifespan compared to mice fed beef tallow [62–64]. These findings coincide with reductions of autoantibodies and circulating immune complexes. Elegant studies from the Fernandes laboratory have related the delayed onset and decreased severity of renal disease exhibited in fish oil-fed NZBWF1 mice to reduced IL-1β, TNF-α, TGFβ1, ICAM-1 and fibronectin expression and increased expression of antioxidant enzymes [65–69]. The ameliorative effects of ω-3 PUFAs have been similarly replicated in two other murine lupus models, BXSB/MpJ and MRL-1pr/1pr, as evidenced by decreased plasma proinflammatory cytokines, proteinuria, and glomerular injury as well as increased lifespan [70, 63, 71–73]. In a recent study comparing the effects of consuming ω-3 PUFA-, ω-6 PUFA- and ω-9 MUFA-rich diets on spontaneous AD development in NZBWF1 mice, we observed elevated plasma autoantibodies, proteinuria and glomerulonephritis in mice fed the latter two diets [24]. In contrast, all three endpoints were markedly attenuated in mice consuming the ω-3 PUFA diet, which contained primarily DHA.

Current Western diets predominantly supply ω-6 PUFA but are deficient in ω-3 PUFA [61]. LA, the primary ω-6 PUFA in plant-derived oils, and its metabolite ARA are major PUFA in phospholipids of cell membranes. By replacing these, DHA is readily integrated into plasma membrane phospholipids in cells throughout the body, including those that mediate immune function [17]. Consistent with this paradigm, we observed dose-dependent increases in DHA and decreases in ARA in lung, kidney and RBCs. DHA was selected for this study over other ω-3 PUFA such as EPA because it has been shown to be superior in preventing spontaneous autoimmunity in NZBWF1 mice [74]. The DHA-rich microalgal oil employed in this study is free of heavy metals and organic pollutants that might be encountered in oils produced from farmed and wild cold-water fish [19]. As this product is formulated to be a source of DHA, the ω-3 PUFA EPA is not present. Accordingly, the microalgal oil employed here is preferable to fish oil-derived formulations because experimental results are attributable to DHA and not confounded by the presence of EPA or other ω-3 PUFAs.

Representative ω-3 PUFA intake recommendations for healthy people range from 0.5 to 2 g/d, but higher levels of consumption up to 20 g/d have been used in clinical therapy trials for established lupus [75–77]. The 0.4, 1.2, and 2.4% dietary DHA concentrations used in this study account for 0.9, 2.7, and 5.4% of total energy intake. Upon extrapolation, a human eating 2000 kcal/d (8.368 MJ/d) would require 2, 6, and 12 g/d to correlate with the amounts consumed in this study. Consequently, in terms of energy percentage in a typical human diet, the concentrations employed here span a range attainable through diet, supplement consumption or by prescription. Therefore, given that some beneficial effects occurred at all three DHA doses, our findings have physiologic relevance to humans. During the course of this study, the European Food Safety Authority deemed that human consumption of supplements containing up to 5 g/d ω-3 PUFA derived from microalgal oil is considered safe [78]. Future perspectives of this model should therefore focus on effects of consuming 5 g/d DHA or lower human equivalents (i.e. ≤ 2.4% of total energy intake) and consider effects of DHA consumption by lupus-prone mice during early life-stages on long-term susceptibility to environmental AD triggers.

## Conclusions

Our findings reveal for the first time that DHA consumption dose-dependently suppresses cSiO_2_ triggering of autoimmunity in female NZBWF1 mice as manifested in the lung, blood and kidney (Fig 14). These observations provide a foundation for further mechanistic exploration of how modulation of the lipidome may be used to prevent or delay triggering of AD by cSiO_2_ and potentially other respirable toxicants. Such knowledge possibly could lead to development of practical, low-cost preventative strategies to reduce the risk of developing AD in cSiO_2_-exposed individuals or even slow progression of existing autoimmunity [79]. Future studies will focus on understanding the mechanisms by which DHA and/or its metabolites suppress cSiO_2_-triggered autoimmunity in this model emphasizing the alveolar macrophage. It will be particularly interesting to determine if unique lipidome signatures are predictive of protective effects of ω-3 PUFA on populations exposed to environmental AD triggers.

## Supporting Information

S1 Table Concentrations of major fatty acids in lung at termination. (DOCX)

S2 Table Concentrations of major fatty acids in red blood cells at termination. (DOCX)

S3 Table Concentrations of major fatty acids in kidney at termination. (DOCX)

## Abbreviations

AD: autoimmune disease
AIN: American Institute of Nutrition
ALA: α-linolenic acid
ANOVA: analysis of variance
ARA: arachidonic acid
BAFF: B cell activating factor
BALF: bronchoalveolar lavage fluid
CON: control diet
cSiO_2_: crystalline silica
DHA: docosahexaenoic acid
DHASCO: microalgal oil containing 40%
DHA: docosahexaenoic acid
ELT: ectopic lymphoid tissue
EPA: eicosapentaenoic acid
FAME: fatty acid methyl esters
GLC: gas liquid chromatography
H&E: hematoxylin and eosin
IL-6: interleukin-6
LA: linoleic acid
lupus: systemic lupus erythematosus
MCP-1: monocyte chemoattractant protein-1
MUFA: monounsaturated fatty acid
NZBWF1: New Zealand Black White F1
OPN: osteopontin
PASH: Periodic Acid Schiff and hematoxylin
PI: post-instillation
PUFA: polyunsaturated fatty acid
TNF-α: tumor necrosis factor-alpha
VEH: Vehicle
