# Correction: Silica-Triggered Autoimmunity in Lupus-Prone Mice Blocked by Docosahexaenoic Acid Consumption

**DOI:** 10.1371/journal.pone.0171877

**Published:** 2017-02-06

**Authors:** Melissa A. Bates, Christina Brandenberger, Ingeborg M. Langohr, Kazuyoshi Kumagai, Adam L. Lock, Jack R. Harkema, Andrij Holian, James J. Pestka

The third author’s name is spelled incorrectly. The correct name is: Ingeborg M. Langohr. The correct citation is: Bates MA, Brandenberger C, Langohr IM, Kumagai K, Lock AL, Harkema JR, et al. (2016) Silica-Triggered Autoimmunity in Lupus-Prone Mice Blocked by Docosahexaenoic Acid Consumption. PLoS ONE 11(8): e0160622. doi:10.1371/journal.pone.0160622

## References

[1] Bates MA, Brandenberger C, Langohr II, Kumagai K, Lock AL, Harkema JR, et al (2016) Silica-Triggered Autoimmunity in Lupus-Prone Mice Blocked by Docosahexaenoic Acid Consumption. PLoS ONE 11(8): e0160622 doi: 10.1371/journal.pone.0160622 27513935 10.1371/journal.pone.0160622 PMC4981380

