# Correction: Silica-Triggered Autoimmunity in Lupus-Prone Mice Blocked by Docosahexaenoic Acid Consumption

**DOI:** 10.1371/journal.pone.0220469

**Published:** 2019-07-24

**Authors:** Melissa A. Bates, Christina Brandenberger, Ingeborg M. Langohr, Kazuyoshi Kumagai, Adam L. Lock, Jack R. Harkema, Andrij Holian, James J. Pestka

In the Animals and diets subsection of the Materials and Methods, there are errors in the first through third sentences of the second paragraph. The correct sentences are: Experimental diets were formulated by modifying American Institute of Nutrition (AIN)-93G diet [25] as summarized in Table 1. All diets contained 64 g/kg food-grade soybean oil and 10 g/kg food-grade corn oil to provide essential fatty acids.

There are a number of errors in Table 1. Please see the correct Table 1 here.

**Table 1:** Compositions of experimental diets.

	Experimental Group			
	CON	0.4% DHA	1.2% DHA	2.4% DHA
Ingredient	(g/kg)			
Casein	184	184	184	184
Dyetrose	121	121	121	121
Cornstarch	366	366	366	366
Sucrose	92	92	92	92
Cellulose	46	46	46	46
t-Butylhydroquinone (TBHQ)	0.01	0.01	0.01	0.01
AIN 93G Salt Mix	32	32	32	32
Ain 93G Vitamin Mix (with vitamin E)	19	19	19	19
L-Cysteine	3	3	3	3
Choline Bitartrate	2	2	2	2
Soybean Oil ^a^^,^^b^	64	64	64	64
Corn Oil^a^^,^^c^	10	10	10	10
High-Oleic Safflower Oil^a^^,^^d^	0	10	30	60
DHA-Enriched Algal Oil^a^^,^^e^	60	50	30	0

^a^ As reported by the manufacturer

^b^ Soybean oil contained 510 g/kg linoleic acid and 226 g/kg oleic acid

^c^ Corn oil contained 612 g/kg linoleic acid and 260 g/kg oleic acid

^d^High-oleic safflower oil contained 750 g/kg oleic acid and 140 g/kg linoleic acid

^e^ Algal oil contained 395 g/kg DHA and 215 g/kg oleic acid
