# Supplementary material for: Silica-Triggered Autoimmunity in Lupus-Prone Mice Blocked by Docosahexaenoic Acid Consumption
Source: PLoS One. 2016 Aug 11;11(8):e0160622. doi: 10.1371/journal.pone.0160622 (PMC4981380; doi:10.1371/journal.pone.0160622)
Supplement: S1 Table — (DOCX) [file pone.0160622.s001.docx]

Table S1- Fatty acid composition of kidney

| Treatment | VEH  CON | *c*SiO_2_  CON | *c*SiO_2_  0.4% DHA | *c*SiO_2_  1.2% DHA | *c*SiO_2_  2.4% DHA |
| --- | --- | --- | --- | --- | --- |
| Fatty Acid | *% of fatty acid in kidney* | | | | |
| 16:0 | 18.79 ± 0.10 | 18.89 ± 0.31 | 20.32 ± 0.36 | 21.99 ± 0.67 | 24.49 ± 1.16 |
| 16:1 (ω-7) *trans* | 0.02 ± 0.001 | 0.02 ± 0.001 | 0.02 ± 0.001 | 0.02 ± 0.001 | 0.03 ± 0.001 |
| 16:1 (ω-7) *cis* | 2.98 ± 0.710 | 2.82 ± 0.620 | 2.07 ± 0.690 | 2.02 ± 0.410 | 3.12 ± 0.800 |
| 18:0 | 12.88 ± 1.29 | 13.64 ± 0.99 | 14.28 ± 1.06 | 12.18 ± 1.03 | 12.90 ± 1.13 |
| 18:1 *trans* | 0.20 ± 0.010 | 0.20 ± 0.010 | 0.21 ± 0.020 | 0.18 ± 0.020 | 0.16 ± 0.010 |
| 18:1 *cis* | 24.64 ± 3.48 | 21.83 ± 2.42 | 15.15 ± 1.13 | 20.74 ± 2.53 | 15.97 ± 1.94 |
| 18:2 (ω-6) | 9.12 ± 0.220 | 9.19 ± 0.390 | 10.27 ± 0.66 | 10.44 ± 0.25 | 7.32 ± 0.490 |
| 20:0 | 0.11 ± 0.001 | 0.10 ± 0.010 | 0.11 ± 0.001 | 0.09 ± 0.010 | 0.10 ± 0.010 |
| 18:3 (ω-6) | 0.05 ± 0.001 | 0.05 ± 0.001 | 0.03 ± 0.001 | 0.02 ± 0.001 | 0.01 ± 0.001 |
| 20:1 (ω-9) | 0.31 ± 0.010 | 0.30 ± 0.010 | 0.25 ± 0.010 | 0.20 ± 0.010 | 0.14 ± 0.010 |
| 18:3 (ω-3) | 0.12 ± 0.020 | 0.11 ± 0.020 | 0.07 ± 0.001 | 0.09 ± 0.010 | 0.07 ± 0.020 |
| 20:2 (ω-6) | 0.18 ± 0.020 | 0.19 ± 0.010 | 0.20 ± 0.010 | 0.13 ± 0.010 | 0.08 ± 0.010 |
| 22:0 | 0.05 ± 0.010 | 0.05 ± 0.001 | 0.06 ± 0.010 | 0.04 ± 0.001 | 0.05 ± 0.001 |
| 20:3 (ω-6) | 0.59 ± 0.060 | 0.61 ± 0.040 | 0.91 ± 0.130 | 0.72 ± 0.100 | 0.30 ± 0.040 |
| 20:4 (ω-6) | 18.10 ± 2.03 | 17.96 ± 1.64 | 11.34 ± 2.32 | 3.44 ± 0.440 | 1.22 ± 0.230 |
| 24:0 | 0.07 ± 0.010 | 0.08 ± 0.010 | 0.09 ± 0.010 | 0.08 ± 0.010 | 0.09 ± 0.010 |
| 20:5 (ω-3) | 0.09 ± 0.010 | 0.10 ± 0.010 | 2.11 ± 0.340 | 5.37 ± 0.710 | 9.42 ± 1.080 |
| 24:1 (ω-9) | 0.06 ± 0.001 | 0.06 ± 0.001 | 0.06 ± 0.010 | 0.05 ± 0.001 | 0.03 ± 0.001 |
| 22:4 (ω-6) | 0.47 ± 0.050 | 0.51 ± 0.080 | 0.05 ± 0.010 | 0.01 ± 0.001 | 0.00 ± 0.000 |
| 22:5 (ω-6) | 0.47 ± 0.050 | 0.55 ± 0.100 | 0.01 ± 0.001 | 0.00 ± 0.000 | 0.00 ± 0.000 |
| 22:5 (ω-3) | 0.29 ± 0.030 | 0.31 ± 0.030 | 0.41 ± 0.020 | 0.46 ± 0.040 | 0.55 ± 0.040 |
| 22:6 (ω-3) | 6.50 ± 0.740 | 6.50 ± 0.460 | 14.13 ± 1.20 | 15.50 ± 1.41 | 18.99 ± 1.59 |
| ∑ SFA | 32.62 ± 1.17 | 33.40 ± 0.93 | 36.69 ± 0.93 | 35.46 ± 0.31 | 39.37 ± 0.81 |
| ∑ MUFA | 29.00 ± 4.13 | 26.10 ± 2.99 | 20.30 ± 2.49 | 24.85 ± 3.17 | 20.22 ± 2.72 |
| ∑ PUFA (ω-3) | 7.11 ± 0.740 | 8.78 ± 1.700 | 17.54 ± 1.82 | 21.51 ± 2.06 | 29.15 ± 2.65 |
| ∑ PUFA (ω-6) | 28.97 ± 2.01 | 29.14 ± 1.52 | 22.94 ± 2.74 | 15.87 ± 1.44 | 8.92 ± 0.740 |
