# Supplementary material for: Silica-Triggered Autoimmunity in Lupus-Prone Mice Blocked by Docosahexaenoic Acid Consumption
Source: PLoS One. 2016 Aug 11;11(8):e0160622. doi: 10.1371/journal.pone.0160622 (PMC4981380; doi:10.1371/journal.pone.0160622)
Supplement: S2 Table — (DOCX) [file pone.0160622.s002.docx]

Table S2- Fatty acid composition of lung

| Treatment | VEH  CON | *c*SiO_2_  CON | *c*SiO_2_  0.4% DHA | *c*SiO_2_  1.2% DHA | *c*SiO_2_  2.4% DHA |
| --- | --- | --- | --- | --- | --- |
| Fatty Acid | *% of fatty acid in lung* | | | | |
| 16:0 | 27.50 ± 0.43 | 40.77 ± 1.14 | 44.32 ± 1.21 | 42.96 ± 0.43 | 45.22 ± 1.31 |
| 16:1 (ω-7) *trans* | 0.04 ± 0.001 | 0.04 ± 0.001 | 0.04 ± 0.001 | 0.04 ± 0.001 | 0.05 ± 0.003 |
| 16:1 (ω-7) *cis* | 7.70 ± 0.260 | 8.42 ± 0.350 | 8.96 ± 0.440 | 9.73 ± 0.290 | 9.24 ± 0.800 |
| 18:0 | 6.40 ± 0.200 | 5.13 ± 0.200 | 4.28 ± 0.130 | 4.12 ± 0.090 | 4.52 ± 0.150 |
| 18:1 *trans* | 0.18 ± 0.010 | 0.11 ± 0.010 | 0.09 ± 0.010 | 0.09 ± 0.001 | 0.13 ± 0.050 |
| 18:1 *cis* | 35.87 ± 0.77 | 23.35 ± 1.03 | 20.90 ± 1.03 | 21.60 ± 0.55 | 19.96 ± 1.14 |
| 18:2 (ω-6) | 6.37 ± 0.190 | 4.97 ± 0.160 | 4.84 ± 0.230 | 4.72 ± 0.070 | 3.15 ± 0.120 |
| 20:0 | 0.07 ± 0.002 | 0.04 ± 0.004 | 0.03 ± 0.001 | 0.03 ± 0.002 | 0.03 ± 0.001 |
| 18:3 (ω-6) | 0.11 ± 0.004 | 0.09 ± 0.005 | 0.06 ± 0.002 | 0.04 ± 0.001 | 0.02 ± 0.001 |
| 20:1 (ω-9) | 0.38 ± 0.008 | 0.24 ± 0.018 | 0.18 ± 0.009 | 0.14 ± 0.007 | 0.09 ± 0.005 |
| 18:3 (ω-3) | 0.11 ± 0.008 | 0.08 ± 0.003 | 0.07 ± 0.006 | 0.07 ± 0.003 | 0.04 ± 0.003 |
| 20:2 (ω-6) | 0.14 ± 0.002 | 0.12 ± 0.005 | 0.09 ± 0.006 | 0.07 ± 0.002 | 0.04 ± 0.002 |
| 22:0 | 0.04 ± 0.001 | 0.03 ± 0.002 | 0.03 ± 0.002 | 0.03 ± 0.001 | 0.02 ± 0.003 |
| 20:3 (ω-6) | 0.37 ± 0.011 | 0.31 ± 0.020 | 0.36 ± 0.020 | 0.25 ± 0.006 | 0.13 ± 0.005 |
| 20:4 (ω-6) | 4.83 ± 0.150 | 5.27 ± 0.498 | 1.97 ± 0.130 | 0.59 ± 0.016 | 0.22 ± 0.006 |
| 24:0 | 0.06 ± 0.002 | 0.05 ± 0.002 | 0.05 ± 0.003 | 0.05 ± 0.002 | 0.06 ± 0.002 |
| 20:5 (ω-3) | 0.03 ± 0.002 | 0.04 ± 0.001 | 0.41 ± 0.030 | 0.69 ± 0.018 | 1.06 ± 0.089 |
| 24:1 (ω-9) | 0.09 ± 0.003 | 0.10 ± 0.003 | 0.09 ± 0.005 | 0.08 ± 0.002 | 0.07 ± 0.008 |
| 22:4 (ω-6) | 1.66 ± 0.071 | 1.52 ± 0.064 | 0.17 ± 0.016 | 0.03 ± 0.002 | 0.01 ± 0.002 |
| 22:5 (ω-6) | 0.29 ± 0.018 | 0.43 ± 0.031 | 0.01 ± 0.001 | 0.01 ± 0.001 | 0.01 ± 0.001 |
| 22:5 (ω-3) | 0.24 ± 0.008 | 0.23 ± 0.011 | 0.52 ± 0.019 | 0.57 ± 0.021 | 0.59 ± 0.012 |
| 22:6 (ω-3) | 1.39 ± 0.043 | 1.44 ± 0.030 | 5.89 ± 0.210 | 7.09 ± 0.123 | 9.65 ± 0.217 |
| ∑ SFA | 35.67 ± 0.54 | 48.26 ± 1.10 | 51.21 ± 1.08 | 50.16 ± 0.75 | 53.62 ± 1.22 |
| ∑ MUFA | 44.92 ± 0.79 | 32.71 ± 0.97 | 30.64 ± 1.03 | 32.05 ± 0.84 | 26.86 ± 1.46 |
| ∑ PUFA (ω-3) | 2.12 ± 0.040 | 2.76 ± 0.210 | 7.07 ± 0.200 | 8.69 ± 0.130 | 11.67 ± 0.27 |
| ∑ PUFA (ω-6) | 13.78 ± 0.23 | 12.51 ± 0.67 | 7.52 ± 0.300 | 5.71 ± 0.080 | 3.58 ± 0.140 |
