# Supplementary material for: Silica-Triggered Autoimmunity in Lupus-Prone Mice Blocked by Docosahexaenoic Acid Consumption
Source: PLoS One. 2016 Aug 11;11(8):e0160622. doi: 10.1371/journal.pone.0160622 (PMC4981380; doi:10.1371/journal.pone.0160622)
Supplement: S3 Table — (DOCX) [file pone.0160622.s003.docx]

S3 Table - Fatty acid composition of RBCs

| Treatment | VEH  CON | *c*SiO_2_  CON | *c*SiO_2_  0.4% DHA | *c*SiO_2_  1.2% DHA | *c*SiO_2_  2.4% DHA |
| --- | --- | --- | --- | --- | --- |
| Fatty Acid | *% of fatty acid in RBC* | | | | |
| 16:0 | 43.69 ± 1.90 | 42.20 ± 2.26 | 40.26 ± 3.06 | 40.50 ± 2.94 | 45.02 ± 2.10 |
| 16:1 (ω-7) *trans* | 0.08 ± 0.010 | 0.08 ± 0.010 | 0.06 ± 0.010 | 0.06 ± 0.004 | 0.05 ± 0.003 |
| 16:1 (ω-7) *cis* | 1.58 ± 0.370 | 2.11 ± 0.440 | 2.45 ± 0.670 | 4.16 ± 0.930 | 3.01 ± 0.960 |
| 18:0 | 21.99 ± 2.30 | 20.54 ± 2.50 | 16.64 ± 2.73 | 12.60 ± 2.12 | 13.78 ± 1.66 |
| 18:1 *trans* | 1.05 ± 0.150 | 1.02 ± 0.230 | 0.99 ± 0.270 | 0.74 ± 0.190 | 0.65 ± 0.140 |
| 18:1 *cis* | 17.56 ± 3.44 | 21.05 ± 3.99 | 24.48 ± 5.23 | 24.41 ± 3.91 | 18.01 ± 3.10 |
| 18:2 (ω-6) | 4.25 ± 1.060 | 4.02 ± 0.650 | 5.88 ± 1.000 | 5.59 ± 0.560 | 4.67 ± 0.320 |
| 20:0 | 0.36 ± 0.020 | 0.33 ± 0.030 | 0.28 ± 0.040 | 0.16 ± 0.020 | 0.17 ± 0.020 |
| 18:3 (ω-6) | 0.00 ± 0.000 | 0.00 ± 0.000 | 0.00 ± 0.000 | 0.00 ± 0.000 | 0.00 ± 0.000 |
| 20:1 (ω-9) | 0.32 ± 0.080 | 0.32 ± 0.050 | 0.34 ± 0.070 | 0.24 ± 0.040 | 0.17 ± 0.020 |
| 18:3 (ω-3) | 0.00 ± 0.000 | 0.00 ± 0.000 | 0.00 ± 0.000 | 0.00 ± 0.000 | 0.00 ± 0.000 |
| 20:2 (ω-6) | 0.00 ± 0.000 | 0.02 ± 0.010 | 0.00 ± 0.000 | 0.00 ± 0.000 | 0.00 ± 0.000 |
| 22:0 | 0.45 ± 0.050 | 0.20 ± 0.050 | 0.10 ± 0.020 | 0.06 ± 0.010 | 0.07 ± 0.010 |
| 20:3 (ω-6) | 0.14 ± 0.040 | 0.20 ± 0.030 | 0.35 ± 0.050 | 0.25 ± 0.030 | 0.16 ± 0.020 |
| 20:4 (ω-6) | 1.63 ± 0.350 | 2.36 ± 0.320 | 1.68 ± 0.250 | 0.73 ± 0.090 | 0.38 ± 0.040 |
| 24:0 | 0.69 ± 0.090 | 0.29 ± 0.060 | 0.17 ± 0.030 | 0.13 ± 0.020 | 0.15 ± 0.030 |
| 20:5 (ω-3) | 0.00 ± 0.000 | 0.00 ± 0.000 | 0.18 ± 0.050 | 0.72 ± 0.110 | 1.40 ± 0.170 |
| 24:1 (ω-9) | 0.41 ± 0.080 | 0.25 ± 0.110 | 0.05 ± 0.010 | 0.03 ± 0.005 | 0.01 ± 0.004 |
| 22:4 (ω-6) | 0.24 ± 0.050 | 0.31 ± 0.050 | 0.07 ± 0.010 | 0.03 ± 0.010 | 0.01 ± 0.004 |
| 22:5 (ω-6) | 0.09 ± 0.020 | 0.13 ± 0.010 | 0.00 ± 0.000 | 0.00 ± 0.000 | 0.01 ± 0.004 |
| 22:5 (ω-3) | 0.00 ± 0.000 | 0.02 ± 0.010 | 0.11 ± 0.020 | 0.19 ± 0.020 | 0.28 ± 0.030 |
| 22:6 (ω-3) | 0.15 ± 0.030 | 0.26 ± 0.030 | 1.36 ± 0.270 | 2.35 ± 0.330 | 5.08 ± 0.620 |
| ∑ SFA | 68.59 ± 4.13 | 65.09 ± 4.74 | 58.95 ± 5.76 | 55.55 ± 4.94 | 61.57 ± 3.50 |
| ∑ MUFA | 21.23 ± 3.60 | 25.09 ± 4.14 | 28.79 ± 5.40 | 30.00 ± 4.55 | 22.22 ± 3.84 |
| ∑ PUFA (ω-3) | 0.15 ± 0.030 | 0.33 ± 0.050 | 1.65 ± 0.340 | 5.31 ± 0.500 | 7.93 ± 0.740 |
| ∑ PUFA (ω-6) | 6.37 ± 1.210 | 7.04 ± 1.030 | 8.06 ± 1.180 | 6.72 ± 0.590 | 5.23 ± 0.340 |
